# Observations show increased nitrate in coastal cloud droplet residuals from large-particle activation and N_2_O_5_ hydrolysis

**DOI:** 10.1126/sciadv.aei3465

**Published:** 2026-10-21

**Authors:** Abigail S. Williams, Lynn M. Russell, Sanghee Han, Veronica Z. Berta, Jeramy L. Dedrick, Teresa Campos, Lauren T. Robinson, Rachel Y.-W. Chang, Ryan N. Farley, Allison C. Aiken, Cynthia H. Twohy, Michael J. Wheeler, Jeremy J. B. Wentzell, John Liggio

**Affiliations:** ^1^ Scripps Institution of Oceanography, University of California San Diego, La Jolla, CA, USA.; ^2^ Atmospheric Chemistry Observations and Modeling Laboratory, National Center for Atmospheric Research, Boulder, CO, USA.; ^3^ Department of Physics and Atmospheric Science, Dalhousie University, Halifax, NS, Canada.; ^4^ Earth and Environmental Sciences Division, Los Alamos National Laboratory, Los Alamos, NM, USA.; ^5^ NorthWest Research Associates, Seattle, WA, USA.; ^6^ Air Quality Research Division, Environment and Climate Change Canada, Toronto, ON, Canada.

## Abstract

Clouds change the chemical composition of aerosols, yet observations characterizing these processes are quite limited. Ground-based in-cloud measurements in coastal Southern California show that the highest nitrate to sulfate ratio (2.5 ± 1.7) in droplet residuals is near cloud base where cloud liquid water content and droplet number concentration are below the observed 20th percentile. The number size distribution of nitrate-rich particles peaks at a larger diameter than sulfate-rich particles, supporting growth to droplets of nitrate-rich particles at lower supersaturations than for sulfate-rich particles. Nitrate mass concentrations are up to three times higher in-cloud compared to pre-cloud and correlated with statistically significant reductions in ozone concentrations, consistent with contributions from in-cloud nitrate formation by N_2_O_5_ hydrolysis. This study provides quantitative relationships of cloud properties to nitrate production in droplet residuals and associated ozone reduction, illustrating the impacts of aqueous reactions in clouds.

## INTRODUCTION

The chemical composition of aerosol particles that serve as cloud condensation nuclei (CCN) influences the activation of those aerosols into cloud droplets, but it can also be altered by the chemical reactions that occur within cloud droplets. Nitrate is a common chemical component of CCN that exists primarily in the form of ammonium nitrate (NH_4_NO_3_) and originates from nitrogen oxide (NO*_x_*) emissions (*1*). During the day, nitrogen dioxide (NO_2_) reacts with hydroxyl radicals (OH) to form highly soluble nitric acid (HNO_3_), which is absorbed by cloud droplets and neutralized by ammonia (NH_3_) to produce aerosol-phase ammonium nitrate. When sunlight is diminished at night, or even diminished within clouds (*2*), NO_2_ is oxidized by ozone (O_3_) to produce photochemically unstable dinitrogen pentoxide (N_2_O_5_). N_2_O_5_ then heterogeneously reacts with the liquid water of cloud droplets (*2*) as well as of aqueous aerosol particles (*3*) to produce HNO_3_ and subsequently particle nitrate. The particle nitrate that remains after droplet evaporation is influenced by the semivolatility of ammonium nitrate, where lower temperatures shift equilibrium toward the particle phase (particle nitrate) and higher temperatures toward the gas-phase (HNO_3_) (*4*). By fostering aqueous reactions, clouds are interconnected to the reactions that control ozone and nitrate concentrations. This connection has important implications for air quality by affecting the tropospheric NO*_x_* cycle and concentrations of tropospheric oxidants, as well as for climate by altering the hygroscopicity of the droplet residuals after evaporation and influencing their ability to act as CCN in future cloud cycles. Despite its importance, the heterogeneous chemistry leading to the formation of nitrate has a wide range of representations and is a source of uncertainty in many air quality models (*5*–*7*). Few observations provide the constraints needed to quantify the chemical interactions of nitrate in clouds.

Measurements of cloud droplet residuals, which are the aerosol particles that remain after collecting and evaporating cloud droplets, have consistently indicated a higher mass fraction of nitrate in residuals compared to out-of-cloud aerosols (*2*, *8*–*12*). Single-particle measurements of droplet residuals have also indicated higher signals of nitrate and higher number fractions of nitrate-rich particles compared to out-of-cloud measurements (*13*, *14*). Measurements of cloud water composition have also shown higher concentrations of nitrate in cloud water samples compared to out-of-cloud air (*15*–*18*). Relative to the amount of sulfate in the droplet residuals, the amount of nitrate has been observed to be lower (*8*, *10*, *13*, *18*–*22*), higher (*2*, *9*, *12*), or both lower and higher (*11*, *17*) depending on the time of the ground-based cloud sampling or time and location of flight sampling. These differences in the relative amount of nitrate and sulfate in cloud droplet residuals have been explained by a difference in aerosol particle size and thus droplet activation efficiency between nitrate and sulfate for two cloud events on a mountaintop in central Sweden. However, the relative particle size associated with nitrate and sulfate is inconsistent among previous studies of CCN and droplet residuals, although repartitioning of nitrate into larger, less acidic particles implies a higher fraction of nitrate in larger particles that can activate to cloud droplets. Specifically, nitrate is observed at larger sizes than sulfate in CCN particles in a flow-through cloud chamber that sampled ambient air in the Netherlands, nitrate is observed at smaller sizes than sulfate in droplet residuals during aircraft flights in Ohio, and no size dependence of nitrate relative to sulfate is observed in droplet residuals at a mountain site in southern China (*2*). This variety of conditions may reflect differences in properties that were not measured or may indicate a gap in our understanding of what role nitrate and sulfate play in activating to droplets in clouds.

In addition to the higher nitrate mass fraction in droplet residuals, higher nitrate mass concentrations in residuals compared to out-of-cloud aerosols suggest an addition of mass through in-cloud processing (*9*). The higher in-cloud nitrate mass concentration (and fraction) is usually attributed to the uptake of HNO_3_ (*9*, *10*, *14*, *17*) or to the heterogeneous hydrolysis of N_2_O_5_ (*2*). Observations linking higher in-cloud nitrate to cloud and environmental parameters are relatively limited and show a variety of results: Higher mass concentrations of nitrate (relative to sulfate) were in droplet residuals near cloud base for airborne measurements off the California coast, lower mass fractions of nitrate were in droplet residuals, and lower mass concentrations of nitrate (relative to sulfate) were in CCN when the ambient temperature was higher for 14 cloud events at a mountain site Germany and a 20-day cloud chamber experiment in the Netherlands (*4*, *9*), and the relative signal intensity of nitrate in droplet residuals was related to variations in temperature, droplet surface area, and concentrations of NO_2_ and O_3_ for 29 cloud events at a mountain site in southern China (*2*).

The conflicting results and limitations quantifying in-cloud nitrate provide the motivation for this study to examine aerosol and cloud measurements taken during the Eastern Pacific Cloud Aerosol Precipitation Experiment (EPCAPE), which occurred at a site located in San Diego in coastal Southern California between February 2023 and February 2024. The EPCAPE measurement site located atop Mount (Mt.) Soledad at an elevation of 251 m ASL is above cloud base height (CBH) about 9% of the time during EPCAPE, encapsulating Mt. Soledad within cloud and allowing in situ cloud observations. The year-long observations sampled a multitude of cloud conditions that allow for a unique evaluation of the factors that control the composition of cloud droplet residuals relative to the ambient aerosol particles out-of-cloud. These results are used to explore how the amount of nitrate in droplet residuals varies because of particle size differences for different microphysical characteristics, the effect of cloud properties on in-cloud nitrate, and the chemical reactions between in-cloud nitrate and ozone concentrations.

## RESULTS

During EPCAPE at Mt. Soledad, a High-Resolution Time of Flight Aerosol Mass Spectrometer (HR-ToF-AMS) characterized the chemical composition of submicron droplet residuals during 352 hours of cloud conditions [cloud liquid water content (LWC) > 0.01 g m^−3^ and visibility < 1 km] and 236 hours of haze conditions (visibility < 5 km and at least one of LWC < 0.01 g m^−3^ or visibility > 1 km), as well as submicron aerosols during 7104 hours of out-of-cloud conditions (visibility > 5 km; see Materials and Methods and table S1). The nitrate mass concentration during out-of-cloud conditions has a mean of 0.32 ± 0.46 μg m^−3^ and increases to 0.43 ± 0.42 μg m^−3^ during cloud conditions (Fig. 1A). This 34% increase is statistically significant (*P* < 0.05) although the cloud mean (0.43 μg m^−3^) falls within the out-of-cloud SD (0.32 ± 0.46 μg m^−3^). In contrast to nitrate, the sulfate mass concentration is higher during out-of-cloud conditions, with a mean of 0.82 ± 0.70 μg m^−3^ that decreases with statistical significance by 56% to a mean of 0.36 ± 0.29 μg m^−3^ during cloud conditions. This results in a statistically significant higher mean nitrate to sulfate ratio of 1.4 ± 0.9 during cloud conditions compared to a mean of 0.3 ± 0.3 during out-of-cloud conditions (Fig. 1B). For haze conditions, the nitrate to sulfate ratio is even higher than during cloud conditions with a mean of 2.8 ± 2.1, in addition to the sulfate mass concentration being significantly lower (Fig. 1). To directly compare pre-cloud to in-cloud changes in composition, 26 single-layer in-cloud events (mean duration, 6.8 ± 5.0 hours) with corresponding pre-cloud events (mean duration 5.3 ± 2.4 hours) were identified (see Materials and Methods and tables S1 and S2). The pre-cloud events are a subset of out-of-cloud conditions and show similar trends in nitrate and sulfate concentrations as the out-of-cloud conditions, and the in-cloud events are a subset of and similar to cloud conditions (Fig. 1). These results are consistent with the findings of previous studies, where the fraction and the ratio of nitrate to sulfate are significantly higher in cloud droplet residuals compared to out-of-cloud particles (*2*, *8*–*11*).

**Fig. 1.**
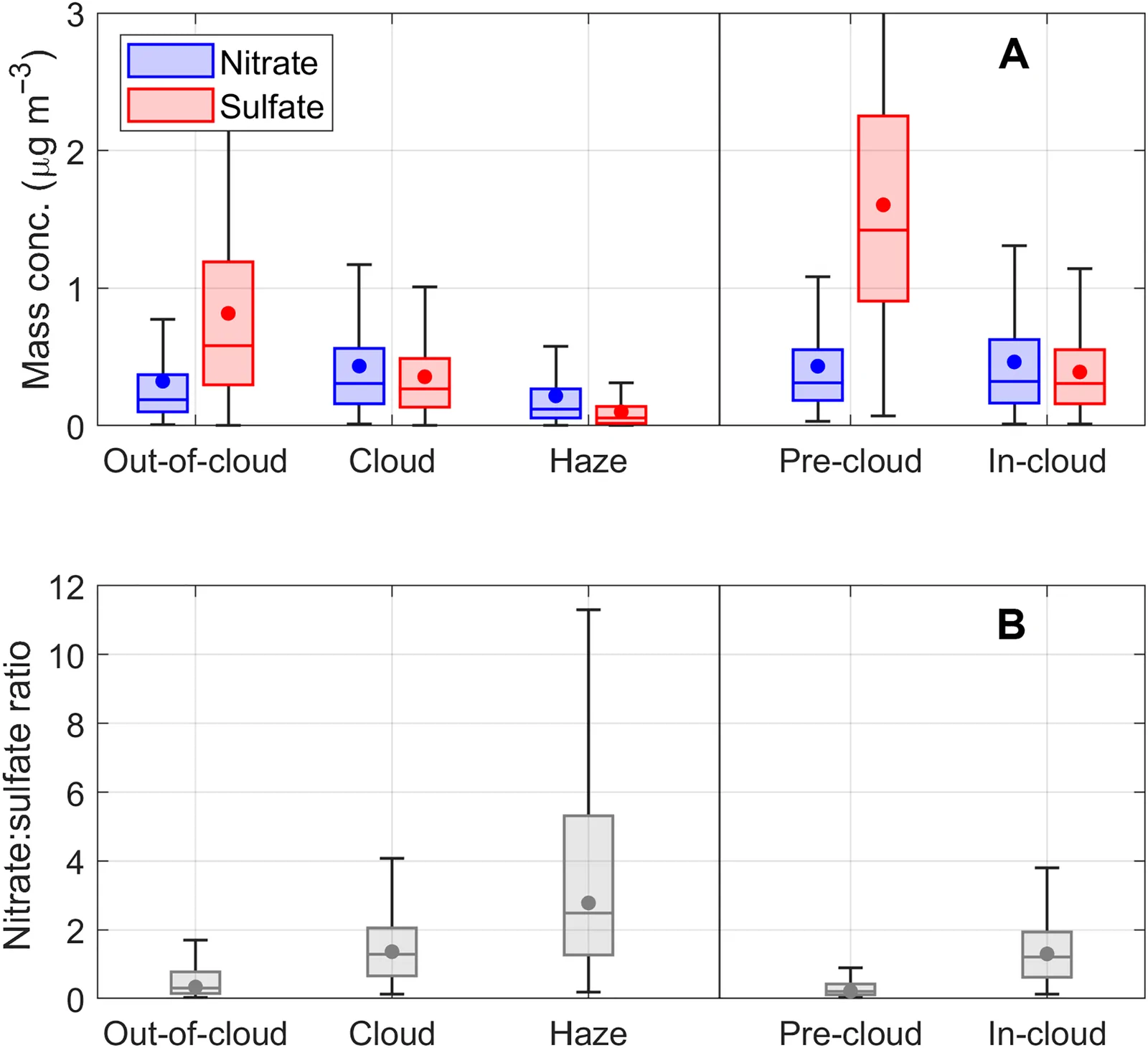
Nitrate and sulfate mass concentrations. (**A**) Box charts of the mass concentration of nitrate (blue) and sulfate (red) separated by out-of-cloud, cloud, and haze conditions and the 26 single-layer pre-cloud and in-cloud events, defined in Materials and Methods. (**B**) Same as (A) but for the ratio of nitrate mass concentration to sulfate mass concentration. On the box, the center line is the median, the lower box edge is the 25th percentile, the upper box edge is the 75th percentile, whiskers extend to the minimum and maximum, and the dot marker is the mean.

### Nitrate in larger particles than sulfate grow to droplets at lower LWC

The chemical composition of the droplet residual population differs from the pre-cloud particle population because not all pre-cloud particles activate into cloud droplets, since only pre-cloud particles with a higher hygroscopicity and larger diameter activate at lower supersaturations. While the hygroscopicity parameters (к) for nitrate (in the form NH_4_NO_3_; к = 0.67) and sulfate [in the form (NH_4_)_2_SO_4_; к = 0.61] are similar with values within 10% of each other, single-particle measurements by the event trigger (ET) mode of the HR-ToF-AMS during EPCAPE indicate that nitrate-rich single particles (fig. S1) are found at larger diameters than sulfate-rich particles during pre-cloud and in-cloud events (Fig. 2, A and B). For the 26 single-layer cloud events, the size distribution of the number fraction of single particles measured during pre-cloud events shows that the mode of nitrate-rich particles peaks at a particle diameter above the nominal HR-ToF-AMS upper diameter cutoff of 1000 nm, while the mode of sulfate-rich particles peaks at a smaller particle diameter of ∼500 nm. These diameters are consistent with previous studies of nitrate and sulfate aerosol distributions, which report a peak in nitrate concentration at 2 to 3 μm and a peak in sulfate concentration at 200 to 600 nm (*23*–*27*).

**Fig. 2.**
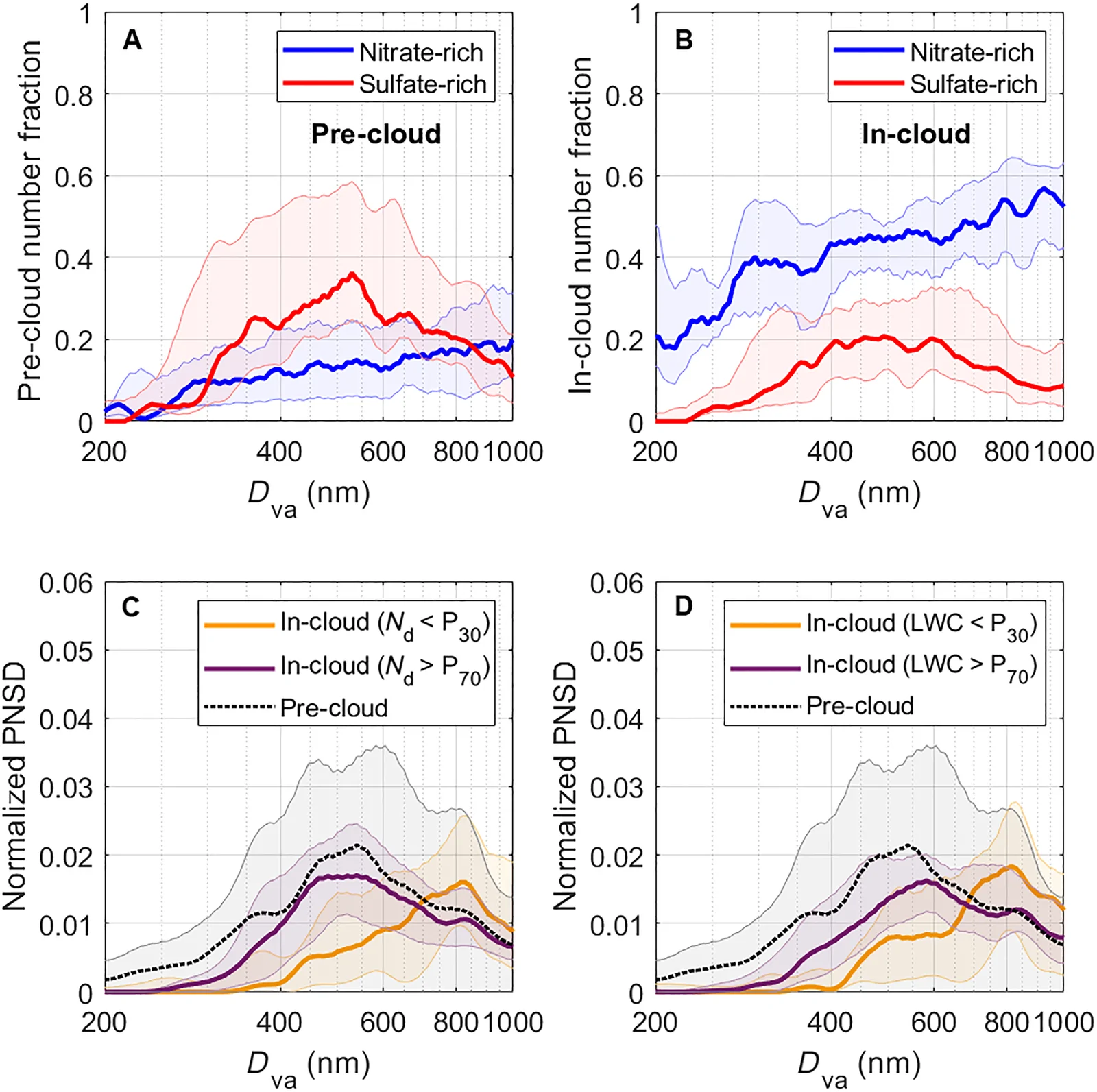
Single PNSDs. (**A**) The median number of nitrate-rich (blue) and sulfate-rich (red) single particles at a particular diameter relative to the sum number of ET single particles measured at that same diameter per pre-cloud event for the 26 single-layer pre-cloud events. Shading represents the 30th and 70th percentiles. (**B**) Same as (A) but for the 26 single-layer in-cloud events. (**C**) The median single PNSD for eight single-layer in-cloud events whose mean droplet number concentration is below 116 cm^−3^ (the 30th percentile, P_30_, of all 26 single-layer in-cloud events), for eight single-layer in-cloud events whose mean droplet number concentration is above 240 cm^−3^ (the 70th percentile, P_70_, of all 26 single-layer in-cloud events), and for all 26 pre-cloud events. Shading represents the 30th and 70th percentiles of the size distribution. (**D**) Same as (C) but for eight single-layer events whose mean LWC is below 0.04 g m^−3^ (the 30th percentile, P_30_, of all 26 single-layer in-cloud events) and above 0.11 g m^−3^ (the 70th percentile, P_70_, of all 26 single-layer in-cloud events).

This trend with particle size is replicated in the single-particle number fractions measured during in-cloud events, with the highest number fraction of nitrate-rich single particles at a diameter at least 500 nm larger than the diameter of the highest number fraction of sulfate-rich particles (Fig. 2B). While the trends with particle size are consistent between pre-cloud and in-cloud, the nitrate-rich number fraction is higher in-cloud (typically 0.2 to 0.6) compared to pre-cloud (typically 0 to 0.2), as seen in Fig. 2B (and fig. S1). The nitrate-rich number fraction is more than two times higher in-cloud than pre-cloud (fig. S1) whereas in-cloud nitrate mass concentration is only 34% higher than pre-cloud (Fig. 1). The difference in the increase of these quantities is likely caused by differences in particle number and mass contributions as well as the mass of nitrate present in the non–nitrate-rich single-particle types. The higher nitrate-rich number fraction in-cloud is associated with a lower number fraction of organic-rich single-particles in-cloud (fig. S2), likely due to the lower hygroscopicity of organic components. The single-particle number size distributions (PNSDs) differ from the mass size distributions of nitrate and sulfate (fig. S3), which is explained by the presence of nitrate in all single-particle types (fig. S1; see Materials and Methods).

For higher cloud LWC and droplet number concentration (*N*_d_), the single particle size distribution of cloud droplet residuals shifts to smaller diameters (Fig. 2, C and D) where more sulfate is found in the pre-cloud particles and droplet residuals (Fig. 2, A and B). For single particles during in-cloud events with mean *N*_d_ and LWC below the 30th percentile, the peak in the number distribution is at a diameter of ∼850 nm. For single particles during in-cloud events with mean *N*_d_ and LWC above the 70th percentile, the peak in the number distribution is at a diameter of ∼500 nm, which is more similar to the pre-cloud distribution. Using 25th and 75th percentile cutoffs shows the same trends as the 30th/70th percentiles, but the 30th/70th percentiles are chosen here to maximize the number of measured single particles in each category. This observed difference in particle diameter is statistically significant and cannot be explained by the higher expected ET sampling rate of the larger particle residuals (see Materials and Methods), suggesting that the in-cloud ET size distributions depend on *N*_d_ and LWC.

In-cloud event means of supersaturation (text S2) (*28*, *29*) are positively correlated to in-cloud event means of *N*_d_ (*r* = 0.70) and LWC (*r* = 0.44; table S3). The mean supersaturation for cloud events with *N*_d_ and LWC above the 70th percentile (Fig. 2, C and D) is higher with statistical significance than the mean supersaturation for cloud events with *N*_d_ and LWC below the 30th percentile (table S3). These observed relationships suggest that for the 26 single-layer cloud events, higher *N*_d_ and LWC are associated with higher supersaturations, particularly for the first 80 m above cloud base (Fig. 3), consistent with the expectation that, as air rises above the lifting condensation level, the supersaturation increases to a maximum in the lower half of the cloud (*30*). Namely, when *N*_d_ and LWC have higher values, indicative of higher supersaturations, particles of smaller diameters can be activated in-cloud (Fig. 2, C and D). This effect is consistent with observations of California coastal clouds, in which higher supersaturations allow for the activation of smaller particles to cloud droplets. Because the measurements of this study are an ensemble of observations across multiple cloud events, the increase in supersaturation, LWC, and *N*_d_ for the first 80 m above cloud base does not necessarily reflect the vertical lifting of a single parcel to a cloud. Rather, these relationships may result from associations across multiple cloud events, for example, with vertically thicker clouds having higher updrafts that cause higher supersaturations or with higher concentrations of pre-cloud particles that cause higher *N*_d_.

**Fig. 3.**
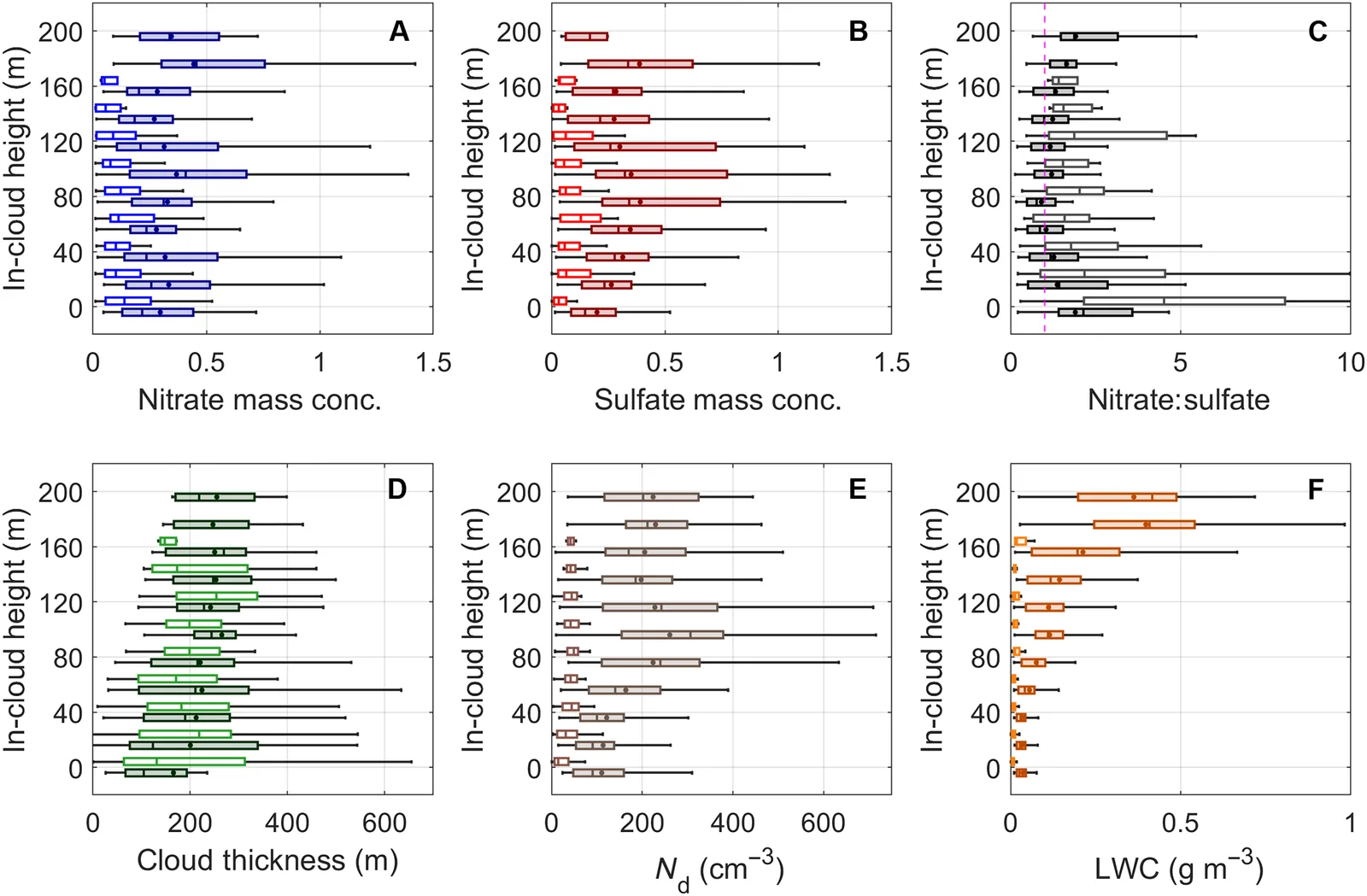
In-cloud vertical profiles. Box plots separated by cloud conditions (filled darker boxes) and haze conditions (unfilled lighter boxes) separated into 20-m bins of the height of Mt. Soledad (251 m ASL) above the observed CBH for (**A**) nitrate mass concentration, (**B**) sulfate mass concentration, (**C**) the ratio of nitrate to sulfate mass concentrations overlaid by the 1:1 line in dashed magenta, (**D**) cloud thickness, (**E**) cloud droplet number concentration, and (**F**) cloud LWC. On the box, the center line is the median, the lower box edge is the 25th percentile, the upper box edge is the 75th percentile, and whiskers extend to the minimum and maximum. The dot marker is the mean for cloud conditions. These in-cloud profiles do not represent the vertical changes in a single cloud but rather the average differences due to changes in the relative height above cloud base at a fixed sampling location.

During cloud conditions at Mt. Soledad, higher values of *N*_d_ and LWC tend to occur at higher in-cloud heights (Fig. 3, E and F), which is defined as the height of Mt. Soledad (251 m ASL) above the cloud base retrieved by radars at Scripps pier (7 m ASL) approximately 3 km northwest of Mt. Soledad. When Mt. Soledad samples at cloud base, the median *N*_d_ and median LWC fall in the lower 20th percentile of all cloud conditions with values of 88 cm^−3^ and 0.03 g m^−3^, respectively, and these conditions are associated with lower cloud vertical thickness (Fig. 3D) as well as shorter cloud event durations (fig. S4). Under these cloud-base conditions, the mean nitrate mass concentration is 0.30 ± 0.23 μg m^−3^, and the mean sulfate mass concentration is 0.20 ± 0.15 μg m^−3^, resulting in the highest ratio of nitrate to sulfate mass concentrations at cloud base with a mean of 1.9 ± 1.2 (Fig. 3C). With increasing in-cloud height (and increasing *N*_d_ and LWC) up to ∼80 m above cloud base, the nitrate to sulfate ratio decreases with statistical significance (by a two-sample *t* test) to a mean value of 1.0 ± 0.8 at 80 m above cloud base, indicating that the nitrate and sulfate mass concentrations become more similar with increasing in-cloud height. This observed in-cloud height trend in the nitrate to sulfate ratio is primarily due to an increasing sulfate mass concentration (Fig. 3B) with in-cloud height, since the nitrate mass concentration remains approximately constant with increasing in-cloud height (Fig. 3A). A similar trend in the nitrate to sulfate ratio is observed during haze conditions, although *N*_d_ and LWC are much lower than during cloud conditions and so the nitrate to sulfate ratio is even higher with a mean of 4.0 ± 2.4 at cloud base (Fig. 3C). Haze conditions occur at in-cloud heights up to 160 m above cloud base (Fig. 3), with the low LWC being partially attributable to variability in CBH between the Scripps Pier and Mt. Soledad producing uncertainty in the in-cloud height but likely also indicating intermittently optically thinner clouds. Figure 4 summarizes the effect of the nitrate and sulfate particle size difference on cloud droplet composition based on the observations during EPCAPE. Nitrate, which is mostly in particles larger than 500 nm during out-of-cloud conditions (Fig. 2A), grows to droplets under conditions of lower LWC and lower *N*_d_ (indicative of lower supersaturations). This nitrate that grows to droplets is associated with a nitrate to sulfate ratio of approximately 3:1 for droplet residuals in haze conditions (Fig. 1B) and a ratio of approximately 2:1 for droplet residuals during in-cloud conditions when Mt. Soledad is near cloud base (Fig. 3C). With increasing in-cloud height and thus increasing *N*_d_ and LWC (indicative of higher supersaturations), a greater number of smaller particles containing sulfate are found in droplets (Fig. 2, C and D), and the sulfate mass concentration increases (Fig. 3B), resulting in a nitrate to sulfate ratio that is near 1:1 (Fig. 3C).

**Fig. 4.**
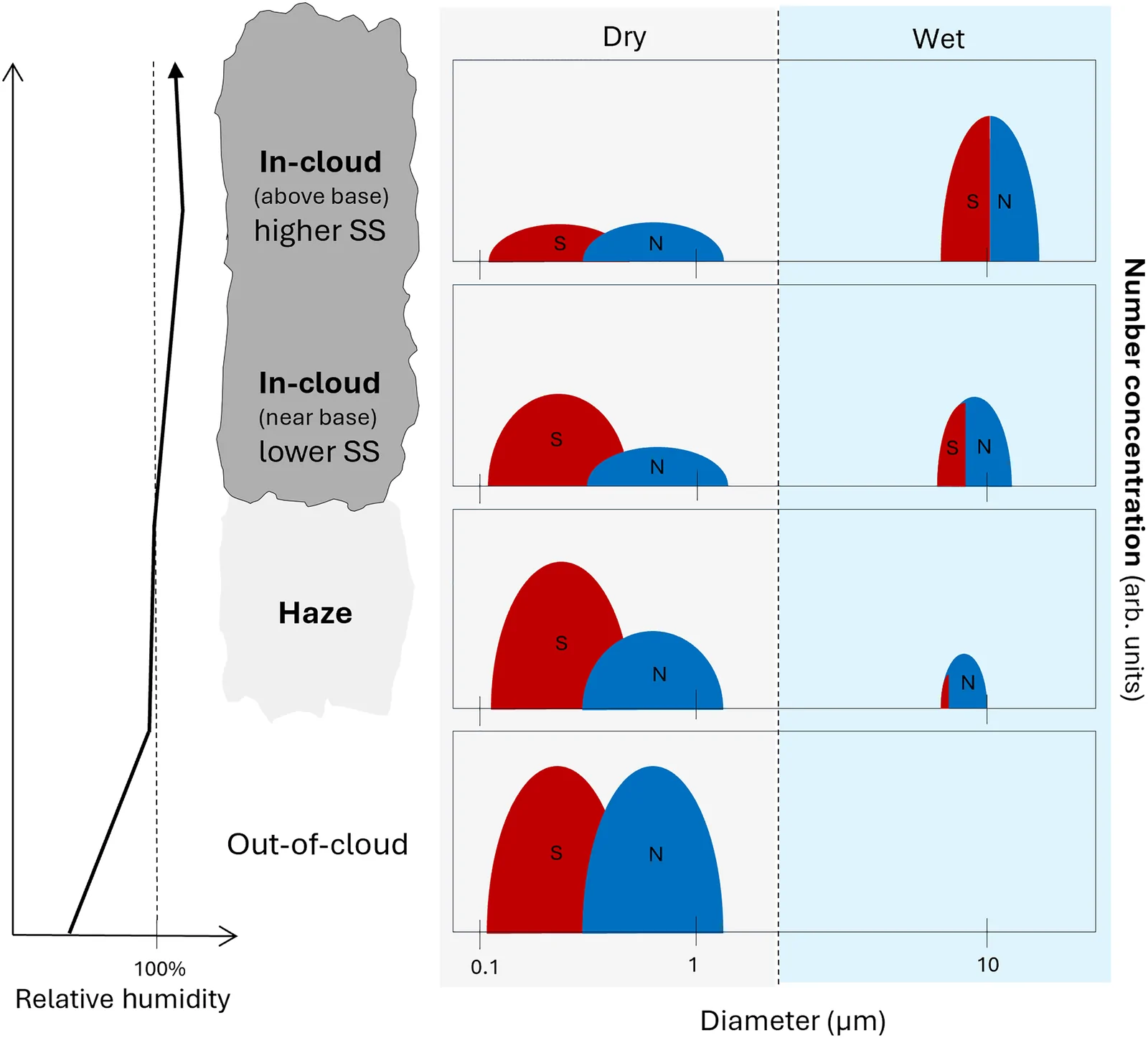
Effects of particle size differences. Graphic summarizing the size difference in nitrate and sulfate particles and its effect on cloud droplet composition. The graphic illustrates changes in the number concentration for both dry particles and GCVI-sampled (wet) particles and does not include cloud top. (**Bottom**) Nitrate is found at larger sizes than sulfate in inactivated particles during out-of-cloud conditions that are subsaturated (RH <100%) as shown in Fig. 2A. (**Bottom middle**) For haze conditions (RH near 100%), the nitrate to sulfate ratio in activated droplets is approximately 3:1 as shown for droplet residuals in Fig. 1B. (**Top middle**) For in-cloud conditions with lower supersaturations (SS; RH > 100%), the nitrate to sulfate mass ratio in droplets is approximately 2:1 as shown for droplet residuals near cloud base in Fig. 3C. (**Top**) For in-cloud conditions with higher supersaturations, the nitrate to sulfate mass ratio in droplets is approximately 1:1 as shown for droplet residuals at higher in-cloud heights in Fig. 3C.

The EPCAPE nitrate to sulfate ratio observations are consistent with model simulations showing that the nitrate to sulfate mass ratio in central California coastal clouds is highest at cloud base and decreases with increasing in-cloud height, which was explained by the rapid uptake of HNO_3_ and relatively slower oxidation of SO_2_ within cloud droplets (*18*). Such in-cloud processing effects on the nitrate concentration during EPCAPE are further examined in the following sections. In-cloud aqueous-phase reactions may also contribute to sulfate concentrations. The increasing sulfate mass concentration with in-cloud height for 0 to 80 m above cloud base (Fig. 3B) corresponds to a decrease in cloud droplet effective diameter (fig. S4). This increase in sulfate mass concentration would also be consistent with the increased rate of aqueous-phase sulfate formation expected in smaller droplets. Smaller droplets have higher surface area to volume ratio, providing higher oxidant concentrations and typically more acidic conditions that increase SO_2_ solubility (*31*). However, SO_2_ mean and median concentrations measured at Scripps Pier were consistently below 0.1 parts per billion (ppb) during EPCAPE (fig. S5). Also, local clouds did not have significant correlations to sulfate formation relative to upwind cloud vertical fraction (*32*). Both the low measured SO_2_ and the dependence of sulfate on upwind clouds suggest that SO_2_ was oxidized before reaching Scripps Pier. For the cloud event observations at Mt. Soledad, in-cloud mean sulfate mass concentrations never exceeded pre-cloud mean sulfate concentrations (fig. S6). As a result, there are no available measurements that would support a quantification of the sulfate formation in clouds on site.

### Cloud properties contribute to increased nitrate concentrations in cloud droplet residuals

For 16 of the 26 single-layer cloud events, the in-cloud mean nitrate mass concentration exceeds the pre-cloud mean nitrate mass concentration by up to a factor of three (Fig. 5), suggesting an addition of nitrate mass through in-cloud processing in more than half of the single-layer cloud events. The remaining events show a decrease in the mean nitrate mass concentration from pre-cloud to in-cloud, and the following analysis includes all observed pre-cloud to in-cloud changes in nitrate for all 26 single-layer cloud events. Of nitrate, sulfate, organics, and ammonium, which together account for >90% of the submicron mass during EPCAPE (*33*), nitrate is the only component to exhibit a statistically significant increase in mean mass concentration from pre-cloud to in-cloud (fig. S6). The pre-cloud to in-cloud change normalized by the pre-cloud concentration is defined as ΔNitrate (text S1) and is correlated to in-cloud event-means of cloud thickness, *N*_d_, LWC, temperature, ozone, and regional NO_2_ (Fig. 5). The ΔNitrate correlations are largely driven by changes in the in-cloud mean nitrate mass concentration (in the numerator of ΔNitrate) rather than by the pre-cloud mean nitrate mass concentration (the denominator of ΔNitrate; table S4). By subtracting and normalizing to the pre-cloud mean nitrate mass concentration in the calculation of ΔNitrate (text S1), some of the in-cloud variability in upwind sources (reflected by variability in the pre-cloud concentrations) is removed. Consequently, ΔNitrate shows a stronger correlation to local conditions than do the in-cloud nitrate mass concentrations.

**Fig. 5.**
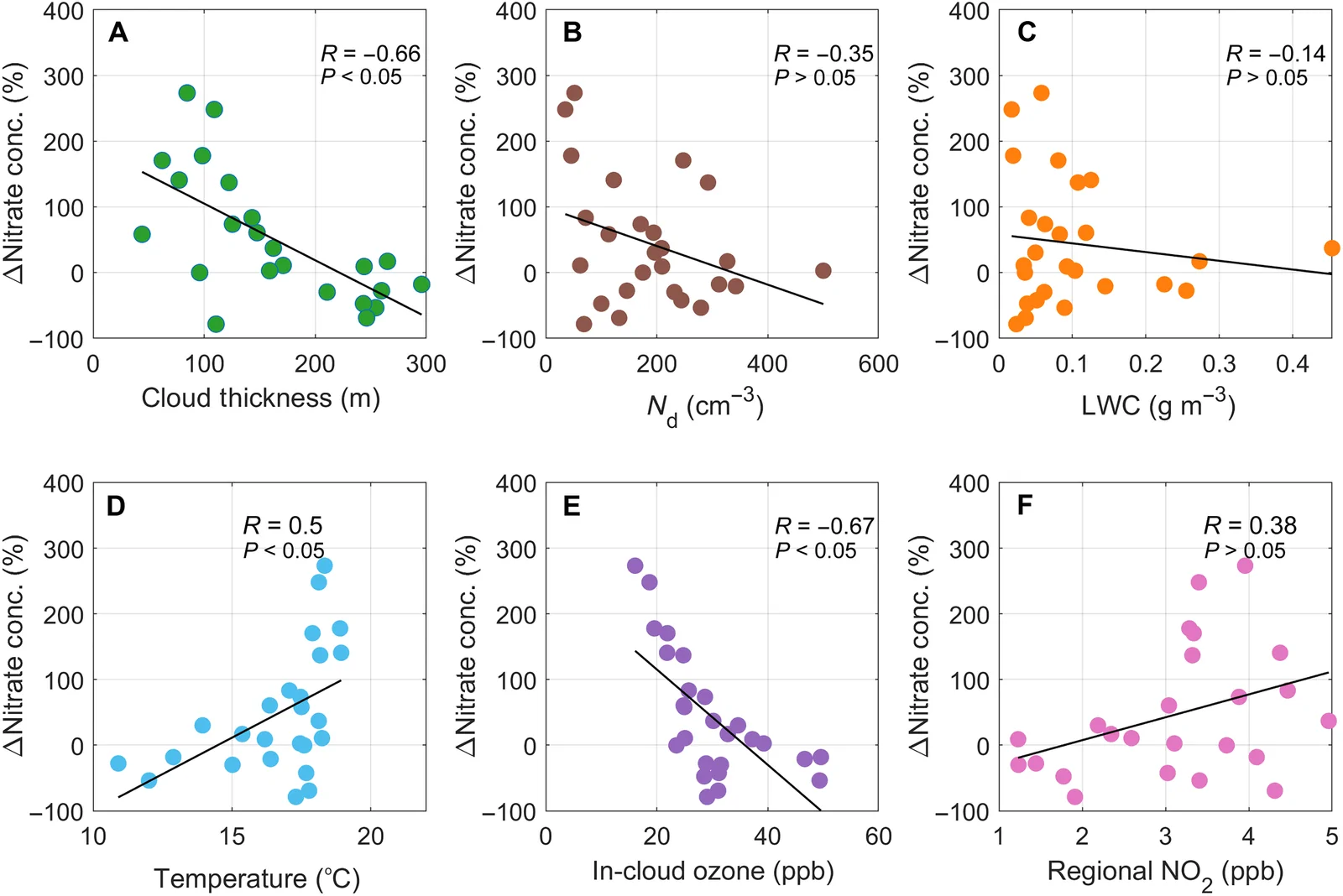
ΔNitrate correlations to environmental variables. Scatterplots for the percent change in mean nitrate concentration from pre-cloud to in-cloud for the 26 single-layer cloud events against (**A**) mean cloud thickness, (**B**) mean droplet number concentration, (**C**) mean LWC, (**D**) in-cloud mean temperature, (**E**) in-cloud mean ozone concentration, and (**F**) mean NO_2_ concentration measured at Kearny Mesa during in-cloud events at Mt. Soledad. Overlaid is the linear regression line, correlation coefficient, and *P* value. Relative to pre-cloud, positive ΔNitrate values indicate higher in-cloud mass, negative values indicate lower in-cloud mass, and a zero value indicates no change. Because of unavailable or flagged measurements, there are 23 events for cloud thickness and 24 events for NO_2_.

A k-means clustering analysis of Hybrid Single-Particle Lagrangian Integrated Trajectory (HYSPLIT) back-trajectories (see Materials and Methods) indicates that, for the 26 single-layer cloud events, there is no statistically significant difference in mean nitrate mass concentration or ozone concentration associated with different trajectory classes (fig. S7), as most of the cloud events occurred during northwesterly back-trajectories that occurred more than 70% of EPCAPE cloud event months (April to September) (*32*, *33*). In addition, the pre-cloud to in-cloud change in mean nitrate mass or ozone concentrations when a corresponding pre-cloud and in-cloud event belong to different trajectory classes does not have a statistically significant difference from the mean change when corresponding pre-cloud and in-cloud events belong to the same trajectory class. On the basis of this lack of dependence on the different upwind source regions associated with different back trajectories, airmass back-trajectories were not considered as a sufficient explanation for the changes in nitrate and ozone concentrations observed in this study. Furthermore, correlations of pre-cloud to post-cloud nitrate concentrations (*r* = 0.80 and pre-cloud to post-cloud ozone concentrations (*r* = 0.76) make it unlikely that the pre-cloud to in-cloud changes are a result of air mass differences as these shifts from pre-cloud to in-cloud to post-cloud tend to occur over more than a day (figs. S8, A and B, and S14).

Multiple linear regression (MLR), with 15-min resolution measurements of the change in in-cloud nitrate mass concentration relative to its pre-cloud mean (ΔNitrate_15M_; text S1), indicates that cloud thickness, *N*_d_, LWC, temperature, and ozone together can explain 30% of the variability (*r* = 0.55, *R*^2^ = 0.3) in ΔNitrate_15M_ (Table 1 and fig. S9). The remaining variability was not explained by the measurements available. The relative contribution of each variable to the ΔNitrate prediction is the magnitude of its coefficient divided by the sum of the magnitudes of all coefficients (expressed as a percentage). Simple linear regressions between the 15-min resolution variables used in the MLR analysis are shown in fig. S10. Several variables show correlations to each other (fig. S9), so ridge regression was specifically used to handle this multicollinearity.

**Table 1.** MLR model for ΔNitrate_15M_. The ridge regression model using 15-min measurements during the 26 single-layer cloud events. The correlation coefficient (*r*), *r*-squared value (*R*^2^), and mean squared error (MSE) for the model are reported. For each predictor variable, the standardized coefficient (β) and relative contribution are reported. Model: ΔNitrate_15M_ = β_o_ + β_1_(cloud thickness) + β_2_(*N*_d_) + β_3_(LWC) + β_4_(temperature) + β_5_(ozone). *r*: 0.55. *R*_2_: 0.30. MSE: 0.70.

Variable	Standardized coefficient		Relative contribution
(Intercept)	β_o_	6.2 × 10^−17^	0%
Cloud thickness	β_1_	−0.21	−19%
*N*_d_	β_2_	0.22	20%
LWC	β_3_	0.23	20%
Temperature	β_4_	0.13	11%
Ozone	β_5_	−0.34	−30%

ΔNitrate has a weak negative correlation to mean *N*_d_ (*r* = −0.35, *P* > 0.05) and a very weak negative correlation to mean LWC (*r* = −0.14, *P* > 0.05), with the highest values of ΔNitrate occurring in events with low *N*_d_ and LWC (Fig. 4). The higher ΔNitrate values are partially explained by the lower *N*_d_ and lower LWC that are associated with the activation of larger more nitrate-rich particles (Figs. 2 and 3). In contrast, the MLR model indicates a positive relationship between ΔNitrate_15M_ and *N*_d_ and between ΔNitrate_15M_ and LWC (Table 1). The positive relationship from MLR differs from the simple linear correlation to LWC in Fig. 4 but is consistent with the expectation that higher aerosol water contents enhance the formation of particulate nitrate (*33*–*36*). These results suggest that the simultaneous occurrence of both nitrate activation and nitrate formation may confound the relationship of ΔNitrate to *N*_d_ and LWC, as evident in the observed differences between the event-mean correlation and 15-min resolution MLR. The highest values of increased in-cloud nitrate mass concentration compared to pre-cloud tend to occur in events with lower *N*_d_ and lower LWC relative to other events (negatively correlated event-means; Fig. 5), while the amount of increased in-cloud nitrate compared to pre-cloud increases with higher *N*_d_ and higher LWC during events (positively related time series by MLR; Table 1). The simultaneous occurrence of both nitrate activation and nitrate formation is supported by parcel model simulations that showed the measured gas-phase HNO_3_ was insufficient to account for the observed cloud water nitrate (*37*).

Higher values of increased in-cloud nitrate mass concentration also tend to occur in vertically thinner clouds, illustrated by a strong negative correlation between the event-means of ΔNitrate and cloud vertical thickness (*r* = −0.66, *P* < 0.05; Fig. 5A) and supported by a negative relationship indicated in the MLR (Table 1). Lower cloud thickness is associated with higher in-cloud temperatures (*r* = −0.75 for event-means, *r* = −0.60 for 15-min time series), with a minimum in-cloud thickness and maximum in temperature occurring during the summer months of July and August (fig. S11). The summer months are characterized by a peak in the estimated inversion strength (EIS) during EPCAPE (fig. S11), which is a common feature of this coastal Southern California region (*38*). Higher values of EIS indicate stronger temperature inversions that cap the tops of the low-level clouds in this region, and stronger inversions lead to less entrainment at cloud top which is associated with decreases in cloud thickness (*39*). For the relatively small range in temperature over this study (<10°C), the relationship between temperature and cloud thickness likely drives the positive moderate correlation between ΔNitrate and the event-mean temperature (*r* = 0.5, *P* < 0.05). The temperature dependence of gas-particle partitioning of HNO_3_ would result in an inverse relationship, since HNO_3_ volatility increases with temperature and more HNO_3_ would remain in the particle phase at lower temperatures (*40*).

In addition to the 26 single-layer cloud events, 22 multilayer cloud events were also considered (table S2). When including the 22 multilayer cloud events with the 26 single-layer cloud events, the trends of the 26 single-layer cloud events shown in Fig. 5 remain (table S4). The correlation of ΔNitrate to *N*_d_ remains negative but becomes significant (*r* = −0.31, *P* < 0.05), the correlation to LWC remains weak and not significant (*r* = 0.05, *P* > 0.05), and the correlation to cloud thickness remains negative and significant (*r* = −0.43, *P* < 0.05).

### Increased nitrate in cloud droplet residuals associated with decreased ozone

Ozone, which was measured in ambient air during out-of-cloud and in-cloud conditions at Mt. Soledad, has the largest influence on ΔNitrate_15M_ based on MLR with a relative contribution of 30% (Table 1) for the 26 single-layer cloud events. The event-means of ΔNitrate and in-cloud ozone are strongly negatively correlated (*r* = −0.67, *P* < 0.05), indicating that higher in-cloud nitrate is associated with lower in-cloud ozone concentrations (Fig. 5E). NO_2_ is expected to be a driver of nitrate formation, but its measurement in the San Diego region has a weak positive and not significant correlation (*r* = 0.38, *P* > 0.05) to ΔNitrate (Fig. 5F). The weakness of the regional NO_2_ correlation may be caused by spatial heterogeneity since NO_2_ was measured ∼9 km inland and 130 m below the location of Mt. Soledad at a site that was not in cloud. The opposite relationships between ozone-ΔNitrate and NO_2_-ΔNitrate are supported by the inverse relationship for regional NO_2_ to regional ozone measurements during April to November (*r* = −0.56, *P* < 0.05). Adding the 22 multilayer cloud events to the 26 single-layer cloud events retains the significant negative correlation between ΔNitrate and in-cloud ozone and a weak positive although not significant correlation between ΔNitrate and NO_2_ (table S4). Here, the spatial heterogeneity of the multilayer cloud events, noted by differences in cloud base between Scripps Pier and Mt. Soledad, likely confounds this dependence for these more complex meteorological conditions.

There is a moderate positive correlation (*r* = 0.55, *P* < 0.05) between the mean nitrate mass concentration and mean ozone concentration during pre-cloud events (Fig. 6A), consistent with the expected linear relationship between ozone and NO*_x_* oxidation products (*41*). A moderate and positive correlation (*r* = 0.67, *P* < 0.05) is also observed during the out-of-cloud conditions that occur after cloud events (i.e., post-cloud; fig. S8c). However, there is a weak negative correlation between nitrate and ozone during in-cloud events (*r* = −0.22, *P* > 0.05; Fig. 6B) and a moderate and significant negative correlation (*r* = −0.53, *P* < 0.05; Fig. 6C) between ΔNitrate and the normalized change in ozone concentration from pre-cloud to in-cloud (ΔOzone) for the 26 single-layer events, with the in-cloud mean ozone concentration being up to 52% lower than the pre-cloud mean ozone concentration in 23 of the 26 single-layer cloud events (Fig. 6C). These measurements provide observational support for model simulations that have shown decreases in ozone in the presence of clouds (*42*) and indicate that a greater amount of additional nitrate mass concentration in-cloud is associated with a greater decrease in ozone concentration.

**Fig. 6.**
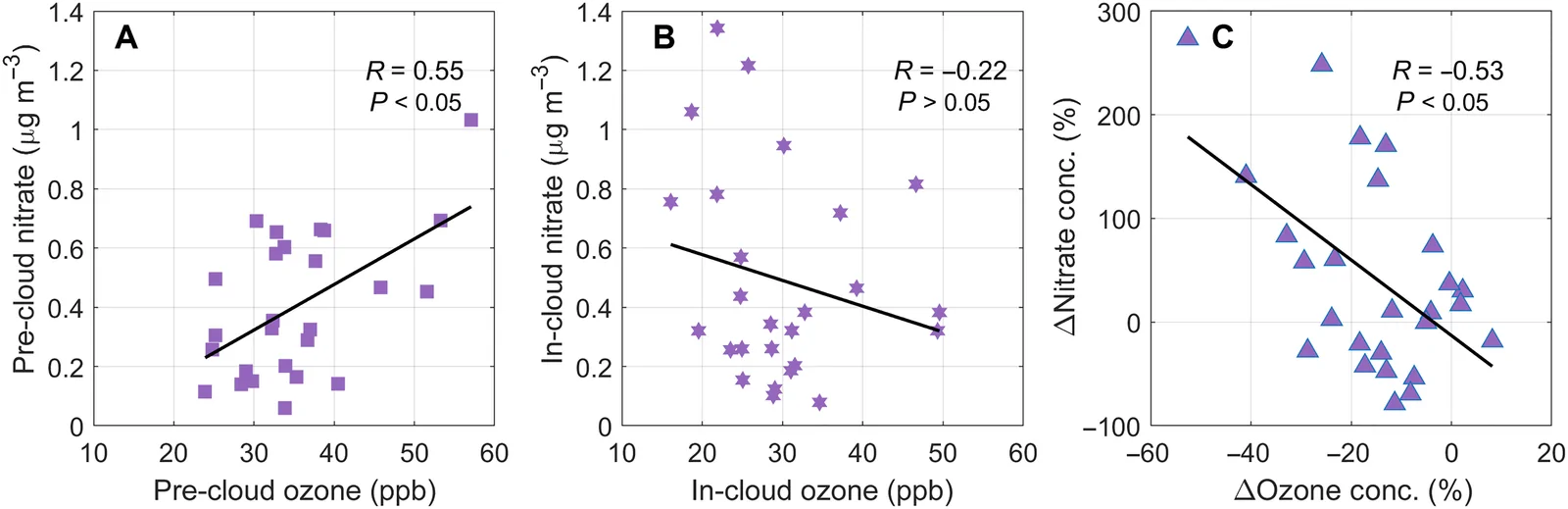
Nitrate and ozone relationships. Scatterplots showing the 26 single-layer cloud event values of (**A**) the mean pre-cloud nitrate mass concentration against the mean pre-cloud ozone concentration, (**B**) same as (A) but for in-cloud events, and (**C**) the percent change from pre-cloud to in-cloud nitrate mass concentration (defined in text S1) against the percent change from pre-cloud to in-cloud ozone concentration. All plots are overlaid with the linear regression line, correlation coefficient, and *P* value.

Figure 7 summarizes how ozone is consumed in the formation of N_2_O_5_, which then reacts with the water in droplets to produce nitrate. This process primarily occurs at night, since the absence of sunlight inhibits further ozone formation and allows for the accumulation of photochemically unstable N_2_O_5_. The absence of sunlight at night also prevents the formation of ozone, making transport the only other possible cause for an increase in its concentration. Ozone decreases throughout the night during both out-of-cloud and in-cloud conditions but decreases more rapidly during cloud conditions (fig. S12). This more rapid ozone decrease is likely partly due to enhanced N_2_O_5_ hydrolysis in cloud droplets, as supported by the negative correlation of a pre-cloud to in-cloud increase in nitrate to decrease in ozone (*r* = −0.53; Fig. 6C). While the observed mean decrease of ΔOzone by 6.5 ppb is too large to be explained by N_2_O_5_ hydrolysis alone given the observed mean increase of 0.28 μg m^−3^ in ΔNitrate, this ΔNitrate measurement by the HR-ToF-AMS includes only the submicron mass concentration after droplet collection by the Ground-based Counterflow Virtual Impactor (GCVI). Additional nitrate in the droplets missed by the GCVI and in supermicron particles produces a larger ΔNitrate than observed, which can account for the observed mean decrease in ozone by N_2_O_5_ hydrolysis (text S3 and table S5). The additional nitrate supports the interpretation of the correlation of ΔNitrate to ΔOzone (Fig. 6C) as reflecting at least a partial attribution of the decrease in ozone to the formation of nitrate from N_2_O_5_ hydrolysis.

**Fig. 7.**
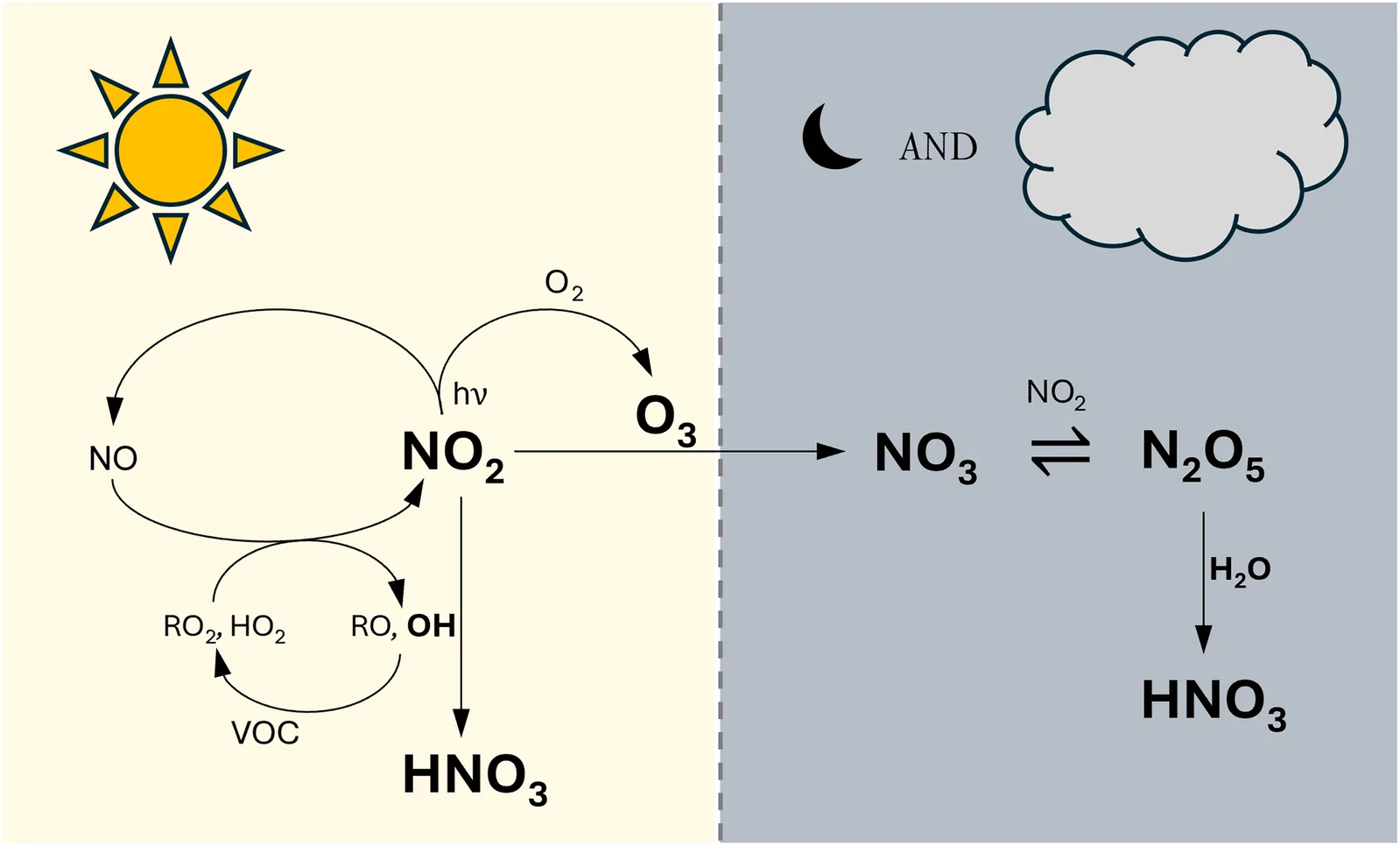
Nitrate formation mechanisms. Schematic of the daytime chemistry (**left**) and nighttime and in-cloud chemistry (**right**) that produces nitrate as HNO_3_. During the day, NO_2_ reacts with OH to form HNO_3_ and NO_2_ is broken down by sunlight (hυ) to form O_3_. Within clouds, similar to at night, NO_2_ reacts with ozone to form photochemically unstable NO_3_ which can then form N_2_O_5_, which then reacts with water to form HNO_3_.

Pre-cloud to in-cloud differences in meteorological variables could also contribute to the observed pre-cloud to in-cloud decrease in ozone. However, the correlations of ΔOzone to the pre-cloud to in-cloud changes in wind direction (*r* = 0.11), wind speed (*r* = −0.13), temperature (*r* = 0.24), boundary layer height (*r* = −0.3), solar radiation (*r* = 0.35), and water vapor mixing ratio (*r* = −0.37) are not statistically significant (*P* > 0.05) and weaker than the statistically significant correlation of ΔOzone to ΔNitrate (*r* = −0.54). While these variables may partially contribute to the observed ΔOzone, their weaker correlations suggest that their contribution to ΔOzone is lower than that of ΔNitrate. ΔOzone is moderately and positively correlated to mean cloud vertical thickness (*r* = 0.53, *P* < 0.05), and cloud thickness is expected to increase with entrainment at cloud top. This correlation is consistent with the largest decrease in ΔOzone occurring when entrainment is weak and the smallest decrease (or even a slight increase) in ΔOzone occurring when entrainment is strong. Stronger entrainment results in more air being mixed into the boundary layer from the free troposphere above, and previous studies have observed entrainment-related increases in boundary layer ozone due to higher ozone concentrations in the free troposphere, where the ozone lifetime is longer (*43*, *44*) and where there is more solar radiation above-cloud for the photochemical production of ozone (*45*, *46*). This means that the addition of ozone into the boundary layer from the free troposphere would weaken the observed decrease in ΔOzone that would otherwise be attributed to ΔNitrate, as observed in some cloud events in which the magnitude of ΔOzone is smaller.

Clouds can significantly attenuate solar radiation such that N_2_O_5_ hydrolysis is observed to be a key pathway of nitrate formation in clouds even during the daytime (*2*). During cloud conditions at Mt. Soledad, the hourly mean downwelling solar radiation measured ∼3 km northwest at the Scripps pier consistently remains below 6 W m^−2^ for all hours of the day, which contrasts to out-of-cloud conditions when the hourly mean can exceed 500 W m^−2^ in the afternoon hours (fig. S13). The reduction in radiation associated with clouds may be sufficient to increase N_2_O_5_ lifetime to support hydrolysis, but since most cloud events at Mt. Soledad begin during nighttime (fig. S14), there were insufficient daytime-only cloud events to disambiguate the cloud-related reductions in radiation from those of nighttime. An increase in ozone after sunrise during cloud conditions suggests that the ozone decrease is most pronounced during nighttime cloud conditions (fig. S12). This interpretation is supported by a weak but significant (*r* = −0.28, *P* < 0.05) negative correlation between the 15-min resolution pre-cloud to in-cloud normalized change in nitrate and ozone concentrations for single-layer cloud events during nighttime hours (fig. S15B), which is consistent with but weaker than the event-mean correlation (*r* = −0.53; Fig. 6C). Weak and not significant correlations were observed between nitrate and ozone for the normalized pre-cloud to in-cloud 15-min measurements during daytime events (fig. S15), for the normalized pre-cloud to post-cloud event-means (fig. S14), for the absolute change in pre-cloud to in-cloud concentrations, and for the normalized pre-cloud to in-cloud event-means during the 22 multilayered cloud events. Overall, the results of this study support an important contribution of N_2_O_5_ hydrolysis to forming nitrate in single-layer coastal clouds at night, accounting for at least part of the lowered ozone concentrations.

## DISCUSSION

Ground-based in-cloud measurements during EPCAPE reveal the ratio of the mass concentration of nitrate to sulfate in cloud droplet residuals is highest at cloud base, with a mean of 2.5 ± 1.7, where *N*_d_ and LWC are relatively low. This ratio decreases with statistical significance with increasing in-cloud height to a value of approximately 1.0 ± 0.8, where the nitrate and sulfate mass concentrations are similar. This composition trend with in-cloud height is a result of increasing *N*_d_ and LWC, which causes a shift in the peak of the measured single PNSD toward smaller particle sizes (from ∼850 to ∼500 nm) where the amount of sulfate is higher. These results indicate that the larger nitrate particles are collected as residuals in low LWC conditions, but the smaller sulfate particles grow to droplets only at higher heights above cloud base where LWC is higher. Given their similar hygroscopicities, the difference in the size distributions of nitrate and sulfate allows for differences in droplet activation depending on the cloud supersaturation (which is positively related to LWC and *N*_d_ for EPCAPE cloud events) and may explain the differences in droplet residual compositions reported in prior studies that did not explicitly characterize the role of cloud water.

The mean nitrate mass concentration for 16 of 26 single-layer in-cloud events exceeds its pre-cloud mean by up to a factor of three, with variations in cloud thickness, LWC, *N*_d_, and temperature each having a relative contribution of 11 to 20% to the prediction of the pre-cloud to in-cloud change in nitrate mass concentration. These changes relative to pre-cloud conditions show increased nitrate uptake at greater cloud thickness, higher LWC, and higher temperature, illustrating how different cloud properties affect cloud droplet chemical composition. This characterization of the important role of cloud properties in determining droplet composition illustrates that nitrate in droplet residuals relative to pre-cloud concentrations is not only affected by the activation of larger particles to cloud droplets but is also affected by in-cloud chemical reactions.

The largest contribution (30%) to the changes between pre-cloud and in-cloud nitrate mass concentrations is associated with ozone in single-layer cloud events, with up to more than a doubling of nitrate mass concentrations from pre-cloud to in-cloud correlated to up to a 52% decrease in ozone concentration from pre-cloud to in-cloud. This decreased ozone is at least partially attributed to forming NO_3_ as a precursor to N_2_O_5_ hydrolysis, which occurs in-cloud in the absence of sunlight. A negative correlation between droplet residual nitrate and ozone in cloud is a reversal of the positive relationship between particle nitrate and ozone out-of-cloud, illustrating that clouds contribute to providing water for the chemical interactions of these copollutants during the night.

Together, these findings illustrate and quantify the multiple effects of cloud properties on increasing nitrate concentrations in droplet residuals relative to out-of-cloud aerosols and an associated decrease in ambient ozone concentrations, which have implications for air quality and climate in regions affected by low-level clouds or fog. The unique and comprehensive nature of these in-cloud observations provide critical constraints for model parameterizations, and this study provides important insights for improving our understanding of nitrate formation in clouds. The observed decrease in ozone concentrations with in-cloud nitrate formation suggests that clouds provide an important sink in the tropospheric ozone budget. Future observational and modeling studies could improve the characterization of these cloud processes for ozone and particle nitrate on air quality in other regions.

## MATERIALS AND METHODS

Measurements during EPCAPE took place between 15 February 2023 and 14 February 2024 at two sites: Mt. Soledad (32.84°N, 117.25°W; 251 m ASL) and the Scripps Pier (32.87°N, 117.26°W; 7 m ASL), which is ∼3 km northwest of Mt. Soledad. Cloud, haze, and out-of-cloud conditions were sampled at the Mt. Soledad site (table S1).

### Cloud conditions and events

At Mt. Soledad, visibility was measured by a Visibility Sensor (Belfort Model 6400) every second. Cloud droplet number concentration (*N*_d_) and LWC were measured by a Fog Monitor (FM-120; Droplet Measurement Technologies, Longmont, CO) for droplets between 2 and 50 μm at 1 Hz. Best estimates of CBH, cloud top height (CTH), and the number of cloud layers at 0.25 Hz were obtained from the Department of Energy (DOE) Atmospheric Radiation Measurement (ARM) Ka-band Zenith Radar Active Remote Sensing of Clouds product (KAZRARSCL) which is based on measurements from a ceilometer, micropulse lidar, and Ka-band zenith radar stationed at the Scripps Pier. Cloud thickness was calculated as the difference between CBH and CTH. The “in-cloud height” at which the Mt. Soledad measurements took place was calculated as the difference between the elevation of Mt. Soledad (251 m) and CBH.

All measurements at Mt. Soledad were averaged to the same 15-min resolution time grid to ensure each grid point had at least two measurements from all instruments. Measurements were then classified as occurring during three conditions (table S1): (i) out-of-cloud conditions when visibility was above 5 km (∼7104 hours), (ii) cloud conditions when visibility was below 1 km and LWC was above 0.01 g m^−3^ (∼352 hours), and (iii) haze conditions when visibility was below 5 km but did not meet cloud condition criteria (∼236 hours). These visibility and LWC thresholds for cloud conditions are similar to that used in other studies (*17*, *22*, *47*–*51*). In addition to the 15-min resolution classification, we identify 48 in-cloud events with corresponding pre-cloud events (table S2). In-cloud events are a subset of the cloud conditions defined above and have FM-120 and HR-ToF-AMS measurements available for at least 1 hour. Pre-cloud events are a subset of the defined out-of-cloud and have HR-ToF-AMS measurements available for at least 1 hour. For all events, the pre-cloud event end time is less than 12 hours before the in-cloud event start time.

Of the 48 cloud events, 22 are multilayer cloud events, while 26 are single-layer cloud events. Mean CBH retrieved from Scripps Pier agrees with cloud conditions at Mt. Soledad (i.e., is below the height of Mt. Soledad) for only 13% (3 of 22) of the multilayer cloud events but 89% (23 of 26) of the single-layer cloud events, suggesting significantly better spatial homogeneity in cloud cover between Scripps Pier and Mt. Soledad during the single-layer cloud events. In addition, the standard deviation in CBH during multilayer cloud events has a maximum of ±4354 m, while the standard deviation during single-layer cloud events has a maximum of only ±65 m, suggesting significantly less variability in cloud structure during the single-layer cloud events. As a result, the analysis of this study focuses on the 26 single-layer cloud events, with results for the multilayer cloud events in the Supplementary Materials. The 26 single-layer events occur between April and November with a mean in-cloud duration of 6.8 ± 5.0 hours and mean pre-cloud duration of 5.3 ± 2.4 hours (table S2), with pre-cloud events occurring on average 3.6 ± 3.0 hours before in-cloud events (fig. S14).

### Particle and gas-phase measurements

At Mt. Soledad, mass concentrations of nonrefractory nitrate, sulfate, organics, ammonium, and chloride were measured every 5 min by a HR-ToF-AMS (Aerodyne Inc., Billerica, MA). When visibility was above 5 km (out-of-cloud conditions), the HR-ToF-AMS sampled submicron aerosol particles behind a PM_1_ cyclone on an isokinetic air inlet. When visibility fell below 5 km (cloud and haze conditions), the HR-ToF-AMS sampled cloud droplet residuals behind a GCVI (Brechtel Manufacturing Inc., Hayward, CA). The HR-ToF-AMS includes a focusing aerodynamic lens with 50% particle transmission at 1-μm vacuum aerodynamic diameter (700 nm in transition-regime aerodynamic diameter for a density of 1.4 g m^−3^) (*52*–*54*), which applies to out-of-cloud as well as cloud and haze conditions. The GCVI collected ∼50% of droplets at a diameter of 9 μm at the GCVI inlet, with *N*_d_ > 9 μm representing 6 to 32% (25th to 75th percentile) of all FM-120-measured droplets and LWC > 9 μm representing 54 to 89% (25th to 75th percentile) of all FM-120-measured droplets. The collected droplets were dried by dilution with warm (40°C) and dry (dew point < 5°C) zero air and directed to the HR-ToF-AMS (bypassing the PM_1_ cyclone). The GCVI 9-μm lower cutoff for droplet diameters meant that the dried submicron particles measured in haze and cloud conditions were a subset of the out-of-cloud dried submicron particles (table S5), making all components but nitrate lower in mass concentration in the droplet residuals than in the out-of-cloud particles (fig. S6).

Corrections for airbeam, particle size, ionization efficiency, and relative ionization efficiency were applied to all HR-ToF-AMS measurements based on postprocessing of weekly calibrations (*33*, *55*). A monthly averaged collection efficiency (CE) for the HR-ToF-AMS was calculated by comparing the HR-ToF-AMS mass concentrations to mass concentrations derived from out-of-cloud PNSDs from a Scanning Electrical Mobility Spectrometer and an Aerodynamic Particle Sizer (SEMS-APS) that were merged (10 nm to 10 μm) after correcting for refractory mass concentrations from a single-particle soot photometer and x-ray fluorescence analysis of submicron filters. The monthly out-of-cloud CE factors ranged between 0.4 and 0.9 and were applied to both out-of-cloud (PM_1_) and GCVI measurements, since the less than 50 hours of GCVI measurements available per month for most months was insufficient to represent average composition. In-cloud GCVI measurements were further corrected with 15-min-averaged enhancement factors ranging between 4.9 and 5.0, calculated from GCVI flow rate measurements every 1 s as in the study Shingler *et al.* (*51*).

The HR-ToF-AMS included ET mode for 2 min of every 5-min cycle to measure the mass spectra of individual particles during all three conditions (out-of-cloud, cloud, and haze). ET measurements were processed, and k-means were clustered using Tofware, the Clustering Input Preparation Panel, and the Clustering Analysis Panel as described by Lee *et al.* (*56*). For each particle measured, the algorithm provides the vacuum aerodynamic diameter (*D*_va_) of the particle, the number of ions of nitrate, sulfate, organics, ammonium, and chloride in the particle, and the cluster (representing a single-particle type) the particle belongs to. Four clusters were identified and were classified as nitrate rich, sulfate rich, less oxidized organics rich, and more oxidized organics rich based on their primary component (fig. S1). The single PNSDs of nitrate and sulfate (fig. S3) differ from the mass size distributions of nitrate and sulfate (fig. S3), for which the accumulation mode peaks are broad but consistent with the number size distributions from a mobility spectrometer for an effective particle density of 1.4 g cm^−3^. While this apparent inconsistency is within the uncertainties of the single-particle and mass size distributions, it may be explained by the distinction between nitrate-rich and the other single particle classifications (fig. S1) being sufficiently obscured by the presence of nitrate in all single particle types to make the mass distributions indistinguishable.

The ET mode of the HR-ToF-AMS primarily measures submicron particles larger than 200 nm (fig. S16), which for the 26 single-layer cloud events represents ∼13% of the submicron ambient particle number during pre-cloud events and ∼39% of the submicron residual particle number during in-cloud events based on merged SEMS-APS PNSD measurements (pre-cloud) and SEMS-only PNSD measurements (in-cloud). ET measurements are indirectly restricted by size based on the minimum ion signals selected for detection, which, for EPCAPE, were set to 4.5 ions per extraction for mass/charge ratio (*m/z*) 41 to 150 (including sulfate, nitrate, and organics), 3 ions per extraction for *m/z* 30 (nitrate), and 2 for *m/z* 36 (chloride). To investigate possible effects of transmission efficiency, the 50% cutoff diameter (*D*_50_) for the HR-ToF-AMS ET measurements was estimated from the ratio of the median ET PNSD to the median SEMS-APS PNSD (fig. S17). The ET effective *D*_50_ is 452 nm during pre-cloud events, above which is ∼2% of the submicron ambient particle number, and 637 nm during in-cloud events, above which is ∼1% of the submicron residual number. For the residual particles sampled by the GCVI, the critical diameter of each single particle was calculated using an estimate of its hygroscopicity (ranging between 0.17 and 0.45) from the ion fractions of the single-particle types (fig. S1) and the estimated effective supersaturation (text S2). A total of 84% of all particles sampled by the GCVI (85% during cloud conditions, 58% during haze conditions) exceed their estimated critical diameter and are expected to have activated. While all residual particles are expected to have grown to droplets large enough to be sampled by the GCVI, the term “activation” is reserved for particles that exceed their critical diameter.

SO_2_ concentrations were measured every 1 s by an ultraviolet (UV) absorbance sulfur dioxide analyzer (model 43i, Thermo Fisher Scientific Inc.) at Scripps Pier. A UV absorbance ozone sensor (model 49C, Thermo Environmental Instruments) continuously sampled on the isokinetic air inlet (i.e., never behind the GCVI) at Mt. Soledad and provided the concentration of ozone every 10 s. Supplemental concentrations of nitrogen dioxide (NO_2_) and ozone at Kearny Mesa, approximately 9 km east of Mt. Soledad at an elevation of approximately 120 m ASL, were reported every 1 hour by the San Diego County Air Pollution Control District and retrieved from their public data archive (*57*).

### Ancillary measurements

Ambient air temperature was measured every 1 min at Mt. Soledad using a Vaisala Weather Transmitter (WXT520; Vaisala Oyj, Finland). Temperature and humidity vertical profiles every 1 hour were obtained from the fifth generation European Centre for Medium-Range Weather Forecasts (ECMWF) reanalysis (ERA5). EIS is used here as a metric of the strength of the temperature inversion, and vertical mixing near cloud top and was calculated from ERA5 reanalysis at the 1000 and 700 hPa levels following the equation by Wang *et al.* (*20*) and Wood and Bretherton (*58*). Downwelling solar radiation measurements every 1 min at Scripps Pier were obtained from the DOE ARM Radiation Data value-added product.

Airmass back-trajectories every 3 hours were obtained from the DOE ARM Trajectories product, which is based on the HYSPLIT model informed by fifth generation ECMWF ERA5 at 0.25° spatial resolution. A k-means clustering analysis based on the geographic coordinates (latitude and longitude) of the trajectories was used to obtain five back-trajectory classes during EPCAPE (*32*, *33*), termed coastal northwesterly (C; from the northwest along the California coast), Los Angeles-Long Beach (L; from the northwest from the urban Los Angeles-Long Beach region), southerly (S; from the south near Tijuana, Mexico), marine westerly (W; from the west over the Pacific Ocean), and easterly (E; from the east).

### Regression analysis

To examine pre-cloud to in-cloud changes in nitrate and its dependence on environmental and cloud variables, we use simple and MLR techniques. Simple linear regressions were performed on the normalized change in mean pre-cloud to mean in-cloud nitrate mass concentration (ΔNitrate; text S1) and event-means of in-cloud variables for each of the identified cloud events. The 15-min resolution time series producing the event-means for the 26 single-layer cloud events are shown in figs. S18 and S19. The Pearson’s correlation coefficient (*r*) is referred to as very strong for *r* > 0.8, strong for 0.6 > *r* > 0.79, moderate for 0.4 > *r* > 0.59, weak for 0.2 > *r* > 0.39, and very weak for *r* < 0.19. A *P* value less than 0.05 is considered significant.

Ridge regression, a form of MLR, was performed using 15-min resolution data during the in-cloud events using the scikit-learn library in Python to address multicollinearity (MLR was not performed on event-means due to the limited sample size of 26). The target (dependent) variable is the normalized change in the 15-min resolution (15M) in-cloud nitrate mass concentration relative to the mean pre-cloud nitrate mass concentration (ΔNitrate_15M_; text S1). The predictor variables are the 15M in-cloud environmental and cloud parameters, excluding NO_2_ since the NO_2_ measurements were not located at Mt. Soledad (and its inclusion did not improve model predictions). Before fitting the regression model, the measurements were *z*-score standardized and split into 70% train and 30% test sets. The optimal regularization parameter (α value) was found on the basis of cross-validation. Since the regression was standardized, the relative contribution associated with each predictor variable is represented by the magnitude of each regression coefficient divided by the sum of the magnitudes of all regression coefficients as in Shen *et al.* (*59*).

## Supplementary Materials

### This PDF file includes:

Texts S1 to S3 Tables S1 to S5 Figs. S1 to S19 References
