# Supplementary material for: Observations show increased nitrate in coastal cloud droplet residuals from large-particle activation and N2O5 hydrolysis
Source: Sci Adv. 2026 Oct 21;12(43):eaei3465. doi: 10.1126/sciadv.aei3465 (PMC13686010; doi:10.1126/sciadv.aei3465)
Supplement: Supplementary file 1 [file sciadv.aei3465_sm.pdf]

Science Advances
AAAS

Supplementary Materials for

Observations show increased nitrate in coastal cloud droplet residuals from large-particle activation and N_2O_5 hydrolysis

Abigail S. Williams *et al.*

Corresponding author: Lynn M. Russell, lmrussell@ucsd.edu

Sci. Adv. **12**, eaei3465 (2026)
DOI: 10.1126/sciadv.aei3465

This PDF file includes:

Texts S1 to S3
Tables S1 to S5
Figs. S1 to S19
References

Text S1. Pre-Cloud to In-Cloud Percent Change Definition

The percent change in mean nitrate mass concentration from pre-cloud to in-cloud is defined as:

$$\Delta Nitrate = \frac{InCloud\ Nitrate_{Mean} - PreCloud\ Nitrate_{Mean}}{PreCloud\ Nitrate_{Mean}} \times 100\% .$$

The 15-minute resolution version of the percent change in nitrate mass concentration from pre-cloud to in-cloud is defined as:

$$\Delta Nitrate_{15M} = \frac{InCloud\ Nitrate_{15M} - PreCloud\ Nitrate_{Mean}}{PreCloud\ Nitrate_{Mean}} \times 100\% .$$

Text S2. Supersaturation Retrieval

Supersaturation was calculated for the cloud events of this study exactly as presented in Dedrick et al (*29*) following the methods described in Dedrick et al (*28*). This method interpolates the droplet number concentration measured by the FM-120 at Mt. Soledad (see Materials and Methods) onto the cloud condensation nuclei (CCN) spectrum measured at 1 hour temporal resolution by a CCN counter at 0.1-1.0% supersaturation at the Scripps Pier. The supersaturation at which the CCN concentration equals the droplet number concentration is taken as the effective supersaturation at the in-cloud height at which Mt. Soledad sampled.

Text S3. Evaluation of Contribution of N_2O_5 Hydrolysis to Ozone Loss and Nitrate Production

Sampling Range of AMS (<1um)

The nitrate observations in this study are measured by an HR-ToF-AMS with an aerodynamic lens that samples submicron particles, with the reported maximum size cut of particles measured by the AMS of 1 µm in vacuum aerodynamic diameter (or 700 nm in transition-regime aerodynamic diameter for a density of 1.4 g/m^3) (*52-54*). There is considerable evidence for additional nitrate in the supermicron range in coastal California that would not be captured by the HR-ToF-AMS (*60, 61*). The HR-ToF-AMS continues to sample submicron particles behind the GCVI in cloud, although the transmission efficiency of the GCVI means that not all drops are delivered to the HR-ToF-AMS. The effect of this under-sampling on concentrations is estimated in Table S5 based on the number concentration transmission but is considered too uncertain to be used to correct the reported values. Since nitrate is semivolatile, the heating of air to 40ºC in the GCVI may also cause some evaporation of particle nitrate. Using the fitted relationship between ambient temperature and sampled nitrate mass during EPCAPE (*33*), the additional mean nitrate mass that may have been lost by GCVI heating is estimated to be 1.48 µg m^{-3} (Table S5) based on the difference between 40ºC and the mean ambient temperature for the 26 cloud events.

Estimated Supermicron Nitrate

Four methods to estimate the amount of nitrate in the supermicron range, obtained from prior publications (*60, 62*), were considered for the cloud events of this study. Number size distributions from merged SEMS and APS measurements (0.01 to 10 µm) during out-of-cloud conditions were converted to mass size distributions assuming particle sphericity and density of 1.4 g/cm^3. Mass concentrations of PM2.5 and PM10 were calculated by integrating the mass size distributions up to 100% cutoff diameters of 2.5 and 10 µm. The first three methods for

estimating the nitrate mass concentration in PM2.5 and PM10 are to assume the supermicron nitrate mass fraction, with the AMS-measured submicron fraction as a lower bound and 1 as an upper bound, as proposed previously for the Los Angeles air basin (*60*). Dried supermicron size distribution measurements were not available during in-cloud conditions, so pre-cloud supermicron concentrations were scaled by the measured submicron in-cloud/pre-cloud ratio to obtain an in-cloud supermicron estimate. As a fourth method, we estimated the amount of nitrate in PM2.5 using the reported PM1/PM2.5 nitrate ratio of 0.77, which is retrieved from AMS, ACSM, and filter measurements between 1998-2022 at multiple sites across the U.S. (*62*). A similar value of ~0.8 is reported for the PM1/PM2.5 nitrate ratio from measurements in Vietnam and China (*63, 64*).

<u>Stoichiometric Ozone Loss for Observed and Estimated Nitrate</u>

As in Ward et al. (*60*), the chemical reactions relating ozone to aerosol nitrate (in the form of ammonium nitrate) are:

$$O_3\,(g) + NO_2\,(g) \rightarrow NO_3\,(g) + O_2\,(g) \quad (1)$$
$$NO_3\,(g) + NO_2\,(g) \rightleftharpoons N_2O_5(g) \quad (2)$$
$$N_2O_5\,(g) + H_2O\,(aq) \rightarrow 2HNO_3\,(aq) \quad (3)$$
$$2HNO_3\,(aq) + 2NH_3\,(g) \rightleftharpoons 2NH_4NO_3\,(aq) \quad (4)$$

Assuming nitrate was formed solely by N_2O_5 hydrolysis (and no other chemical or meteorological factors affect the concentrations), the loss of ozone would be half of the increase in nitrate in moles, which is a maximum loss of 2.8 ppb of ozone. This ozone loss is smaller than the observed mean decrease of 6.5 ppb in ozone from pre-cloud to in-cloud (Table S5). The mean pre-cloud to in-cloud increase in estimated PM2.5 and PM10 nitrate ranges between 0.36 and 18.9 μg m^{-3} (Table S5). In support of these relatively high nitrate concentrations, PM2.5 nitrate in excess of 10 μg m^{-3} was commonly observed in Pasadena and attributed to formation by N_2O_5 hydrolysis (*60*). Based on stoichiometric calculations for each cloud event estimate, the mean pre-cloud to in-cloud increase in PM2.5 and PM10 nitrate mass corresponds to a mean loss of ozone between 0.95 and 19.6 ppb (Table S5), with the observed mean ozone decrease of 6.5 ppb falling within this range.

<u>Constrained Nitrate Production Rate from Measured NO_2 and O_3</u>

The estimated nitrate production rate constrained by on-site O_3 and regional NO_2 measurements shows that attributing the high estimated PM2.5 and PM10 nitrate concentrations to N_2O_5 hydrolysis is possible. Assuming all nitrate radical production is consumed by N_2O_5 hydrolysis, the loss rate of N_2O_5 is equal to the production rate of the nitrate radical described by $P(NO_3) = k[NO_2][O_3]$, where $k = 1.2 \times 10^{-13}\exp(-2450/T)$ molecules cm^{-3} sec^{-1} (*60*). The production rate of nitrate ions is two times the nitrate radical production rate, specifically $2*k[NO_2][O_3]$, which is calculated using the 15-min averages of measured O_3 at Mt. Soledad and regional NO_2 (measured at Kearny Mesa) for the cloud events of this study. The maximum possible nitrate formation solely from N_2O_5 hydrolysis is the integrated production rate over each cloud event duration, which amounts to nitrate mass concentrations between 0.2 and 22.3 μg m^{-3} per event (mean of 5.3 μg m^{-3}; Table S5) for the 26 cloud events (which have a mean duration of 6.8±5.0 hours).

Summary N_2O_5 Hydrolysis Contributions to Ozone Loss

The observed ozone loss is smaller than the calculated ozone loss associated with the estimated supermicron nitrate expected from N_2O_5 hydrolysis and supports the possibility that a substantial fraction of the observed decreases in ozone may be attributable to N_2O_5 hydrolysis. Nonetheless, this calculation only provides bounds on the maximum possible attribution. The primary support for attributing some of the ozone loss to N_2O_5 hydrolysis is the observed moderate and significant correlation between pre-cloud to in-cloud decreases in ozone with increases in submicron nitrate ($r = -0.53$, $p < 0.05$), with weak and not significant correlations of these pre-cloud to in-cloud changes to meteorological variables and airmass sources.

Table S1.

Summary of the criteria for cloud conditions and cloud events utilized in this study. The upper part of the table indicates the details for out-of-cloud conditions, cloud conditions, and haze conditions. The lower part of the table indicates the selection of the 26 single-layer cloud events of this study.

**Cloud Conditions**		
Term	Criteria<br>*Any 15-min point where:*	Number of Hours
**Out-of-cloud conditions**	Visibility > 5 km	7104
**Cloud conditions**	Visibility < 1 km<br>LWC > 0.01 g m^{-3}	352
**Haze conditions**	Visibility < 5 km<br>*and at least one of:*<br>LWC < 0.01 g m^{-3}<br>*or* Visibility > 1 km	236

**Cloud Events**				
Term	Criteria<br>*Any time period where:*	Number of Events	Hours of Cloud	Hours of Haze
All GCVI events longer than 1 hr	Visibility < 5 km (i.e. GCVI on) for at least 1 hr continuously	154	341	199
Non-consecutive events	*In addition to above:*<br>Events within 6 hr merged as same event	95	342	204
Measurements of drop distributions at site	*In addition to above:*<br>FM120 measurements available for entire event duration	86	335	152
Sufficient measurements of cloud composition	*In addition to above:*<br>AMS measurements available for at least 1 hr of cloud condition points during event	50	255	73
Sufficient pre-cloud	*In addition to above:*<br>Pre-cloud conditions measured within 12 hours before event, visibility > 5 km (i.e. GCVI off) for at least 1 hr during event	48	236	71
**Single-layer cloud events**	*In addition to above:*<br>Only a single-layer cloud exists for cloudy points	**26**	**158**	**45**

Table S2.

Summary of the 26 single-layer pre-cloud and in-cloud events. The first column denotes the subplot of Figures S17 and S18. The second to fifth column lists the dates and times in UTC of the pre-cloud and in-cloud events. The sixth and seventh columns indicate the number of hours (rounded down to the nearest 15-min) that meet cloud conditions or haze conditions during each in-cloud event.

	**Pre-Cloud Start Time**	**Pre-Cloud End Time**	**In-Cloud Start Time**	**In-Cloud End Time**	**Cloud Hours**	**Haze Hours**
(a)	08-Apr-2023 17:30	09-Apr-2023 00:00	09-Apr-2023 05:20	09-Apr-2023 22:11	14.75	0.75
(b)	26-Apr-2023 13:45	26-Apr-2023 23:00	27-Apr-2023 01:32	27-Apr-2023 16:53	14.25	0.75
(c)	27-Apr-2023 17:30	27-Apr-2023 23:15	28-Apr-2023 05:07	28-Apr-2023 17:36	9.75	2.5
(d)	09-Jun-2023 04:15	09-Jun-2023 06:45	09-Jun-2023 09:51	09-Jun-2023 12:21	2.25	0
(e)	17-Jun-2023 05:58	17-Jun-2023 11:30	17-Jun-2023 11:47	17-Jun-2023 17:19	3.75	1.5
(f)	01-Jul-2023 03:36	01-Jul-2023 07:45	01-Jul-2023 11:20	01-Jul-2023 15:29	1	2
(g)	01-Jul-2023 22:45	02-Jul-2023 03:15	02-Jul-2023 10:44	02-Jul-2023 17:04	4.25	0.75
(h)	03-Jul-2023 20:30	04-Jul-2023 02:45	04-Jul-2023 03:52	04-Jul-2023 18:09	13	2
(i)	11-Jul-2023 02:30	11-Jul-2023 03:30	11-Jul-2023 14:21	11-Jul-2023 15:58	1.25	0
(j)	11-Jul-2023 23:02	12-Jul-2023 06:30	12-Jul-2023 07:20	12-Jul-2023 14:48	2.75	0.5
(k)	13-Jul-2023 23:38	14-Jul-2023 04:00	14-Jul-2023 06:56	14-Jul-2023 11:17	3	1.25
(l)	14-Jul-2023 21:39	15-Jul-2023 03:30	15-Jul-2023 04:04	15-Jul-2023 09:54	2.75	1.5
(m)	17-Jul-2023 19:37	18-Jul-2023 04:00	18-Jul-2023 07:01	18-Jul-2023 15:24	7.75	0.25
(n)	19-Jul-2023 01:23	19-Jul-2023 05:15	19-Jul-2023 07:02	19-Jul-2023 10:54	1.5	1.75
(o)	24-Jul-2023 05:15	24-Jul-2023 07:30	24-Jul-2023 08:15	24-Jul-2023 11:30	7.25	1.5
(p)	24-Jul-2023 19:30	24-Jul-2023 21:30	25-Jul-2023 00:50	25-Jul-2023 20:28	16.75	1.5
(q)	27-Jul-2023 04:08	27-Jul-2023 09:15	27-Jul-2023 09:44	27-Jul-2023 14:50	3	1.5
(r)	27-Jul-2023 23:04	28-Jul-2023 07:00	28-Jul-2023 09:00	28-Jul-2023 16:55	2.75	4.75
(s)	02-Aug-2023 03:22	02-Aug-2023 07:45	02-Aug-2023 08:49	02-Aug-2023 13:11	1.75	2.25
(t)	06-Aug-2023 02:54	06-Aug-2023 09:15	06-Aug-2023 09:37	06-Aug-2023 15:57	4	2
(u)	06-Aug-2023 23:12	07-Aug-2023 07:00	07-Aug-2023 07:33	07-Aug-2023 15:21	3	1.5
(v)	14-Aug-2023 20:00	15-Aug-2023 00:00	15-Aug-2023 01:55	15-Aug-2023 14:30	11	4.5
(w)	17-Aug-2023 05:15	17-Aug-2023 07:00	17-Aug-2023 13:14	17-Aug-2023 20:08	5.75	1
(x)	30-Aug-2023 17:45	31-Aug-2023 04:15	31-Aug-2023 04:31	31-Aug-2023 15:33	2.25	3.25
(y)	08-Oct-2023 04:30	08-Oct-2023 10:15	08-Oct-2023 16:26	09-Oct-2023 16:44	14.5	5.25
(z)	05-Nov-2023 16:42	05-Nov-2023 22:15	05-Nov-2023 23:38	06-Nov-2023 05:10	3.75	0.75

Table S3.

Values for the correlation coefficient of event-mean supersaturation (SS) to event-mean liquid water content (LWC) or droplet number concentration (N_d) measured by the FM-120 for 19 of the 26 single-layer cloud events, the mean and standard deviation of SS for the cloud events with mean LWC or N_d below the 30th percentile (P_{30}), and the mean and standard deviation of SS for the cloud events with mean LWC or N_d above the 70th percentile (P_{70}).

**Correlation of SS to LWC**	**SS for LWC < P_{30}** (mean±SD)	**SS for LWC > P_{70}** (mean±SD)	**Correlation of SS to N_d**	**SS for N_d < P_{30}** (mean±SD)	**SS for N_d > P_{70}** (mean±SD)
r = 0.44<br>p = 0.06	0.03±0.02 %	0.09±0.03 %	r = 0.70<br>p < 0.05	0.03±0.03 %	0.08±0.02 %

Table S4.

Correlation coefficients as in Figure 5 but instead for the in-cloud minus pre-cloud nitrate mass concentration (numerator of ΔNitrate; Text S1), the in-cloud nitrate mass concentration, the pre-cloud nitrate mass concentration (denominator of ΔNitrate), and ΔNitrate with the inclusion of the 22 multi-layer cloud events (48 cloud events in total).

	**Cloud Thickness**	**N_d**	**LWC**	**Temperature**	**Ozone**	**NO_2**
**In-cloud minus Pre-Cloud Nitrate Mass Conc. (μg m^{-3})**	-0.59	-0.23	0.03	0.41	-0.61	0.37
**In-cloud Nitrate Mass Conc. (μg m^{-3})**	-0.35	-0.01	0.15	0.35	-0.22	0.45
**Pre-cloud Nitrate Mass Conc. (μg m^{-3})**	0.39	0.30	0.18	-0.05	0.54	0.14
**ΔNitrate, 22 multi-layer cloud events included (%)**	-0.43	-0.31	0.05	0.35	-0.45	0.07

Table S5. Pre-Cloud to In-Cloud Changes in Nitrate and Ozone.

Size Range	Method for Obtaining Changes	Nitrate Increase Mean | Max (µg m^{-3})	Ozone Decrease Mean | Max (ppb)
Measured			
PM1	In-Cloud – Pre-Cloud (Schneider et al., 2017 (*9*); Modini et al., 2015 (*11*); Zhang et al., 2022 (*2*))	0.28 | 0.85	6.5 | 17.8
Estimated (accounting for undersampling by GCVI)			
PM1	[a]Correction factor for number fraction of particles not transmitted through GCVI (Shingler et al., 2012 (*51*))	Measured multiplied by: 1.25 | 1.53	
PM1	Semivolatile nitrate mass loss due to GCVI heating (Han et al., 2025 (*33*))	Measured plus: 1.48 | 1.89	
Estimated (accounting for undersampling by HR-ToF-AMS)			
PM2.5	Nitrate PM1/PM2.5 ratio is 0.77 (Li et al., 2025 (*62*))	0.36 | 1.1	1.2 | 3.6
PM10	Nitrate mass fraction >1µm is same as <1µm (Ward et al., 2025 (*60*))	1.2 | 3.8	4.0 | 12.6
PM10	[b]Nitrate is 50% of mass >1 µm	9.6 | 28.0	31.7 | 92.4
PM10	Nitrate is all of mass > 1 µm (Ward et al., 2025 (*60*))	18.9 | 55.4	62.5 | 182.6
Estimated (maximum nitrate yield by N_2O_5 hydrolysis)			
All	Nitrate production rate constrained with on-site O_3 and regional NO_2 measurements	5.3 | 22.3	

Footnotes:
[a]Transmission efficiency is calculated for cloud drop number, and hence this factor is based on residual particle *number*. Transmission efficiency of particle mass is approximate since droplet mass proportional to residual particle mass.
[b]50% of coarse mode is consistent with semivolatile fraction of coastal California measurements (*7*) and is used as an upper bound for this study since it is near the maximum yield of nitrate formed by N_2O_5 hydrolysis with measured O_3 and NO_2 (bottom row of table).

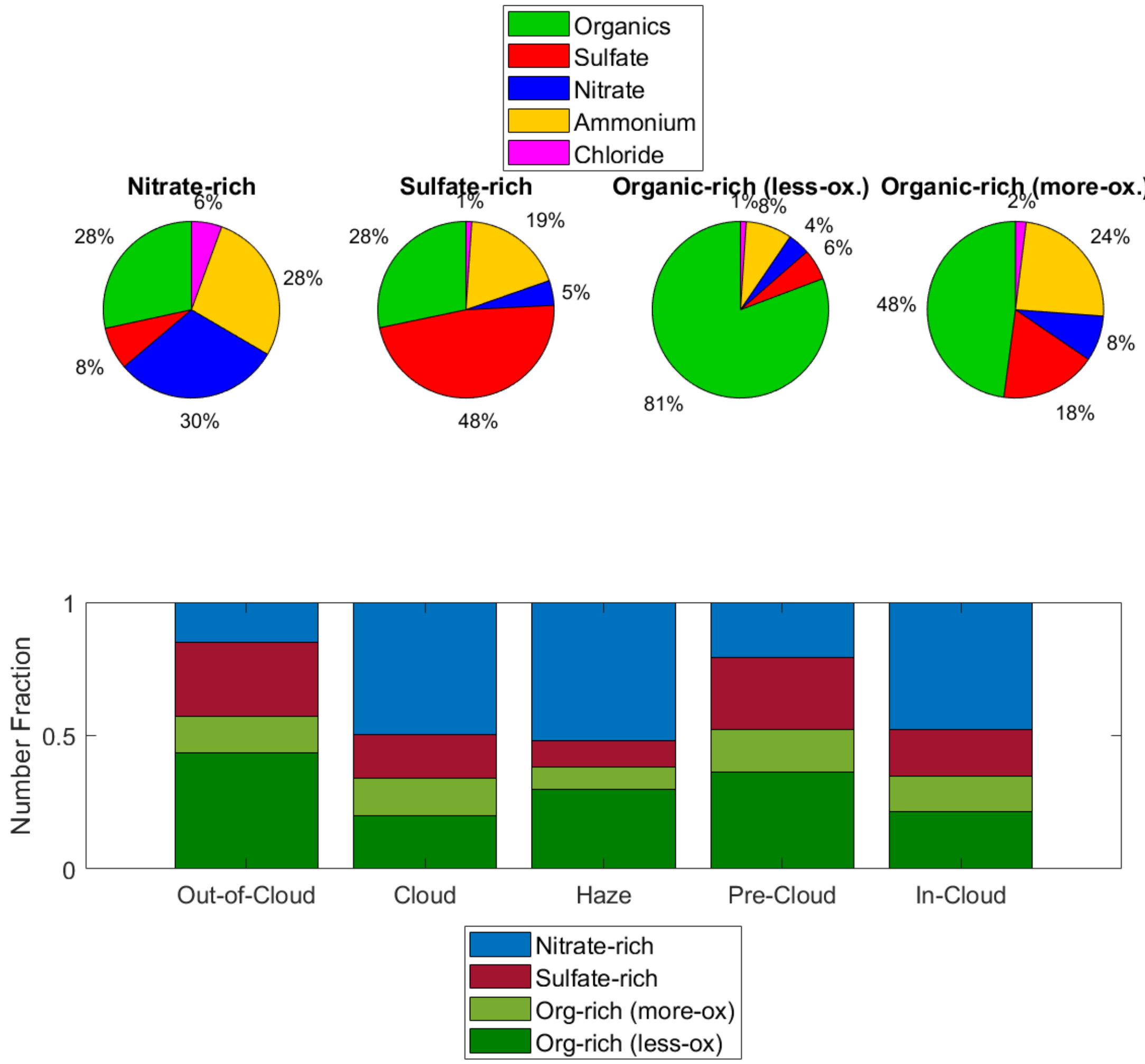

Fig. S1.

(Top) Pie charts of the composition of the four cluster centroids of the ET measurements. Each cluster represents a single-particle type and is named after its primary component. There are two organic-rich types, less-oxidized and more-oxidized, since the former has a relatively higher signal at m/z 43 and the latter has a relatively higher signal at m/z 44. (Bottom) Number fractions (number of a single particle type divided by the sum of all single particles measured) of the four particle types for out-of-cloud, cloud, and haze conditions, and the 26 single-layer pre-cloud and in-cloud events.

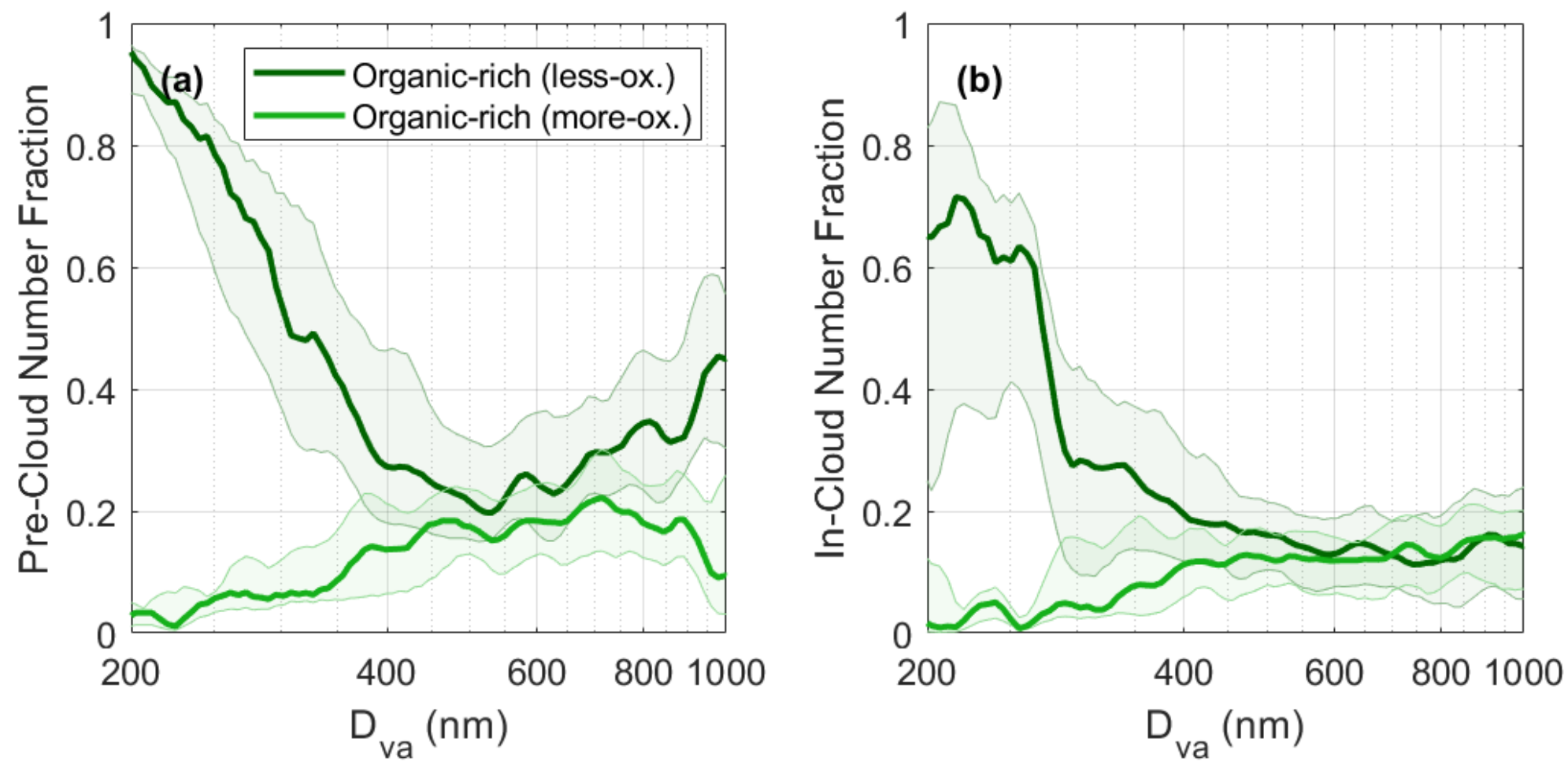

Fig. S2.

(a) The median number fraction of measured single-particles at a particular diameter belonging to the organic-rich (less-oxidized) and organic-rich (more-oxidized) particle types during all 26 single-layer pre-cloud events. Shading represents the 30th and 70th percentiles. (b) Same as (a) but for all 26 single-layer in-cloud events.

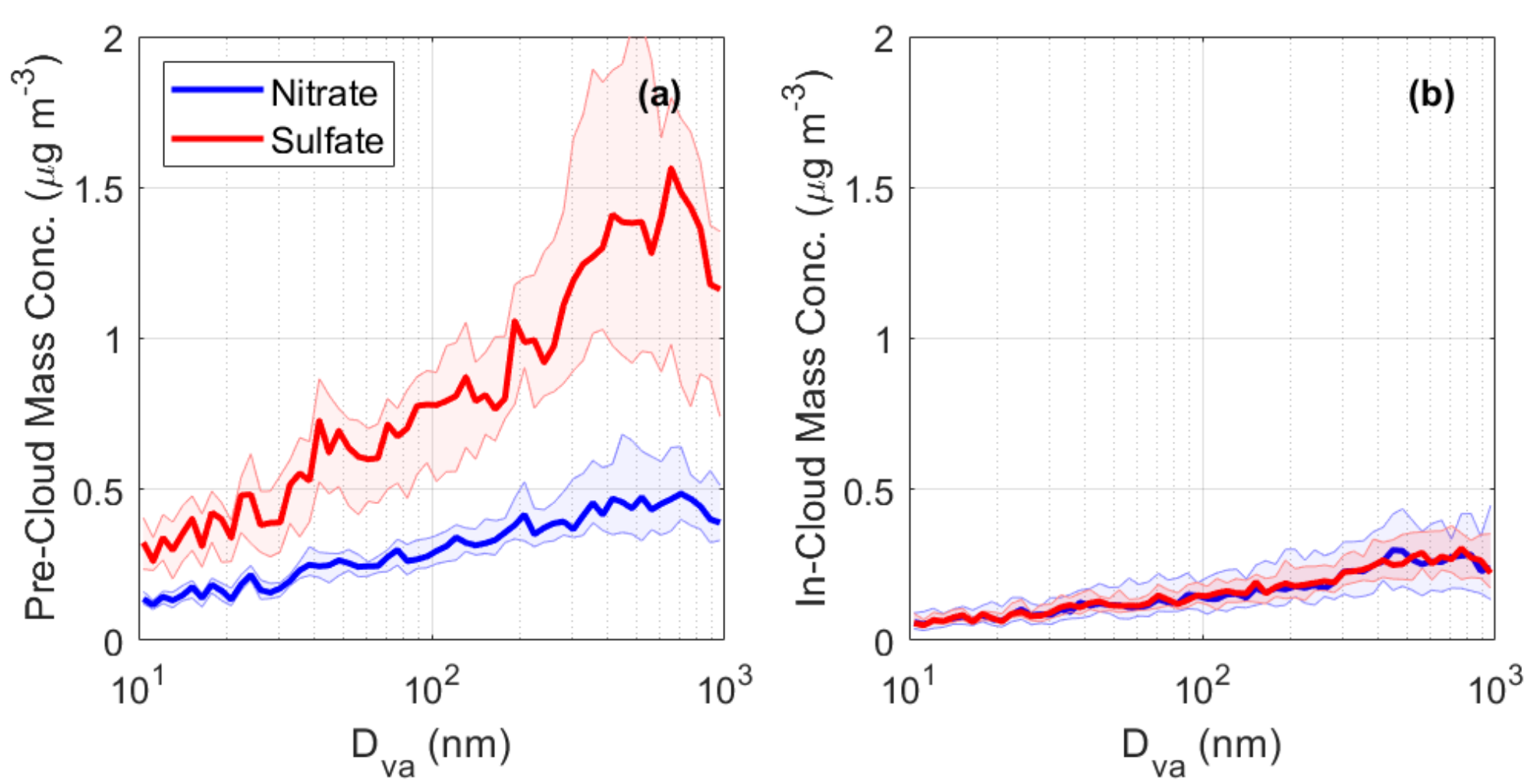

Fig. S3.

Figure S8 (a) size distribution of the mass concentration of nitrate and sulfate from HR-ToF-AMS particle time of flight (PToF) measurements during the 26 pre-cloud events. (b) same as (a) but for the 26 in-cloud events.

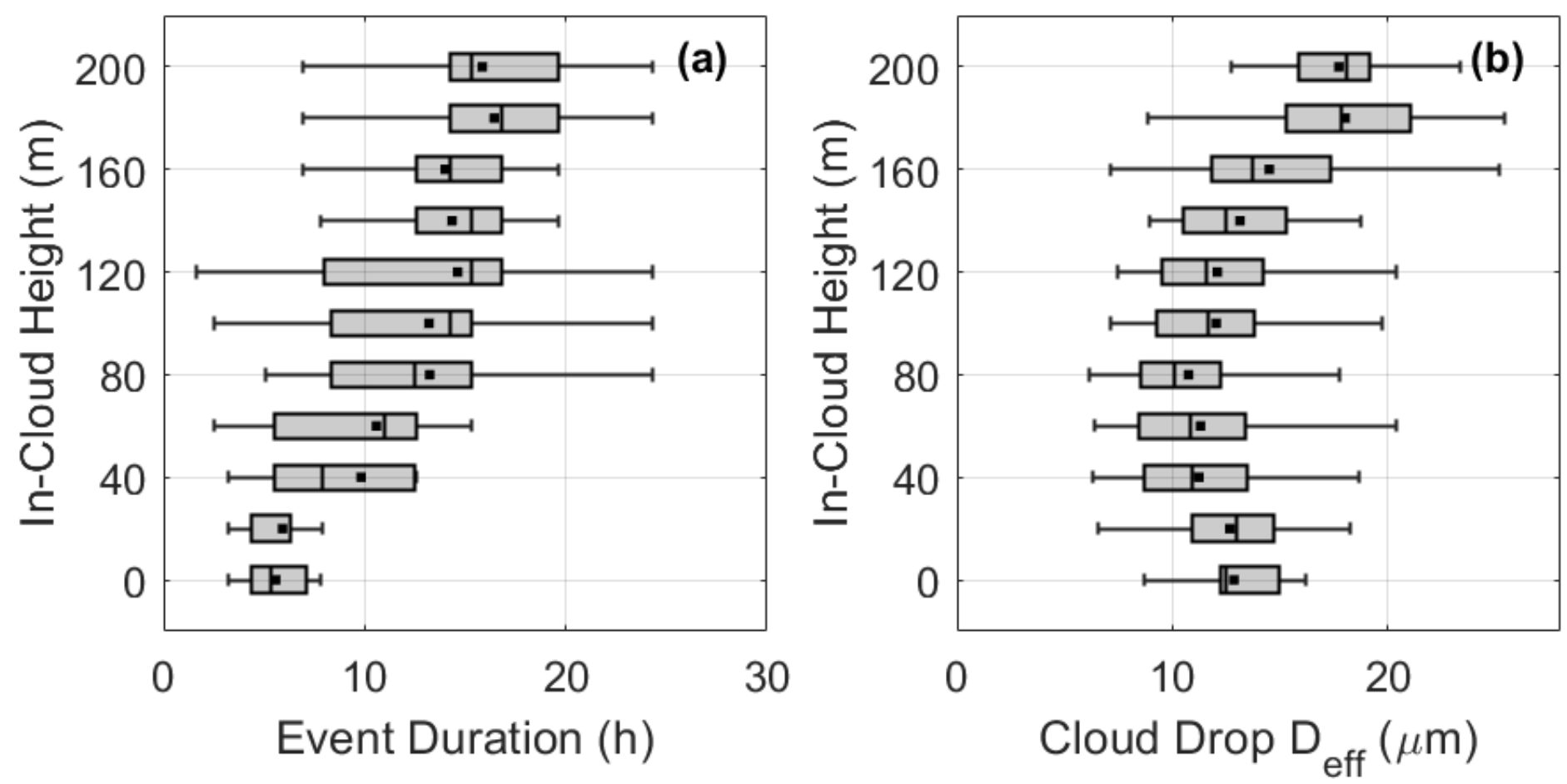

Fig. S4.
Box charts of (a) the in-cloud event duration (duration of the event each 15-minute point belongs to) and (b) cloud droplet effective diameter (D_{eff}) during the 26 single-layer cloud events separated into 20 m bins of the height of Mt. Soledad (251 m ASL) above the observed cloud base height. On the box, the center line is the median, lower box edge is the 25th percentile, upper box edge is the 75th percentile, whiskers extend to the minimum and maximum, and the dot marker is the mean. These in-cloud profiles do not represent the vertical changes in a single cloud, but rather the average differences due to changes in the relative height above cloud base at a fixed sampling location.

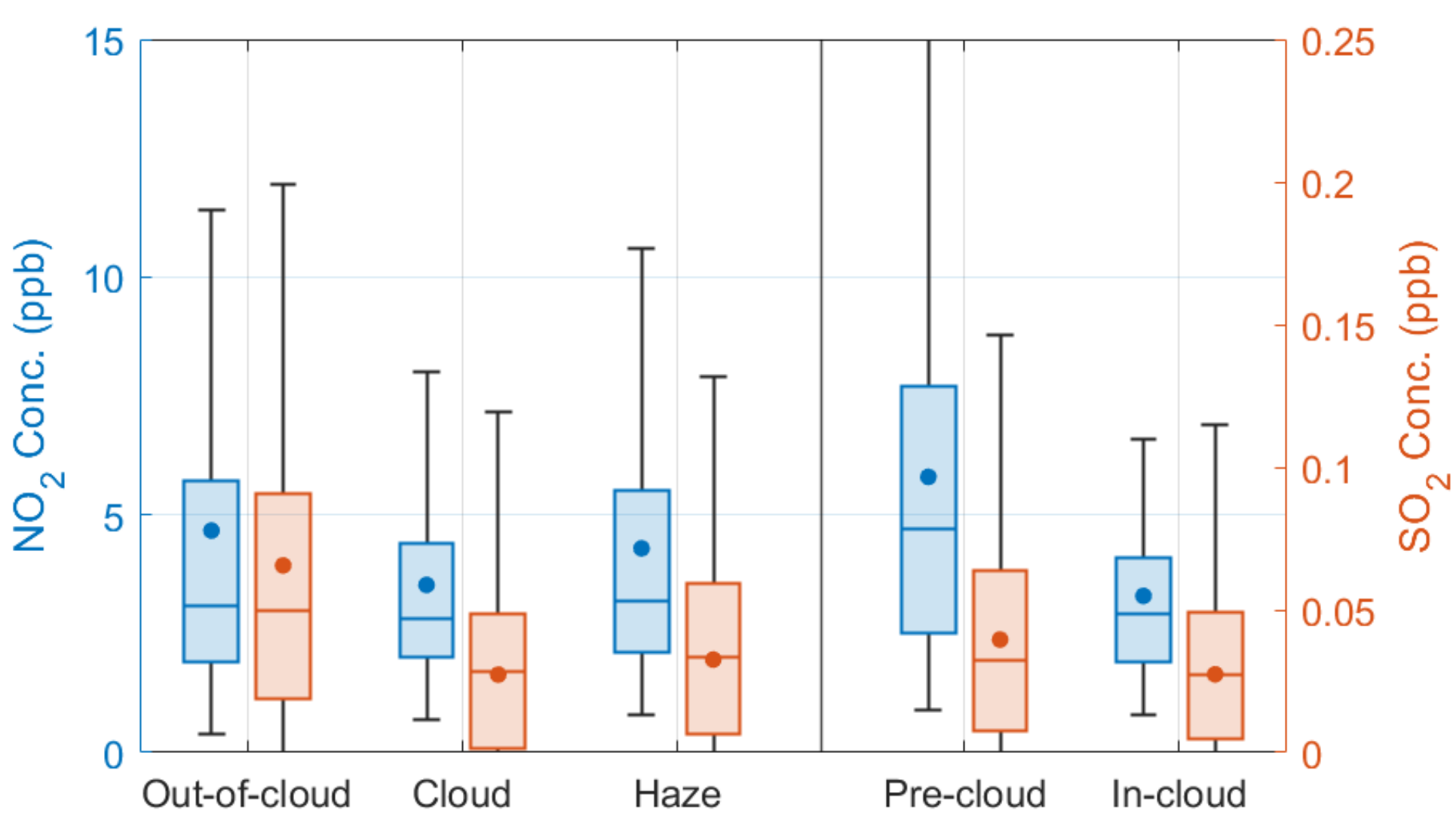

Fig. S5.
Box charts of the concentration of NO_2 (blue) and SO_2 (red) separated by out-of-cloud, cloud, and haze conditions and the 26 single-layer pre-cloud and in-cloud events. On the box, the center line is the median, lower box edge is the 25th percentile, upper box edge is the 75th percentile, whiskers extend to the minimum and maximum, and the dot marker is the mean.

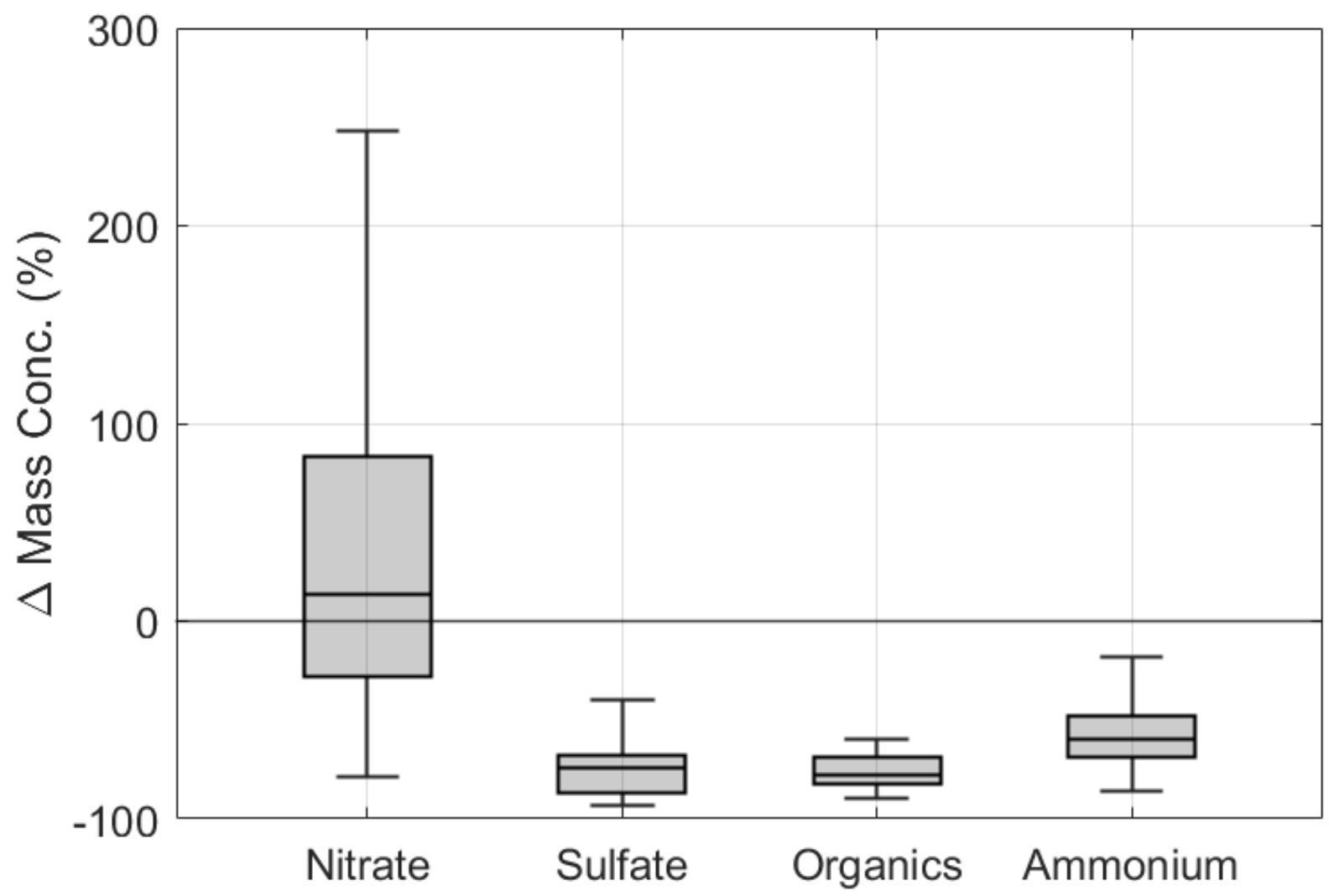

Fig. S6.
Box charts of the percent change in mean mass concentration from pre-cloud to in-cloud (Text S1) for the 26 single-layer cloud events for nitrate, sulfate, organics, and ammonium.

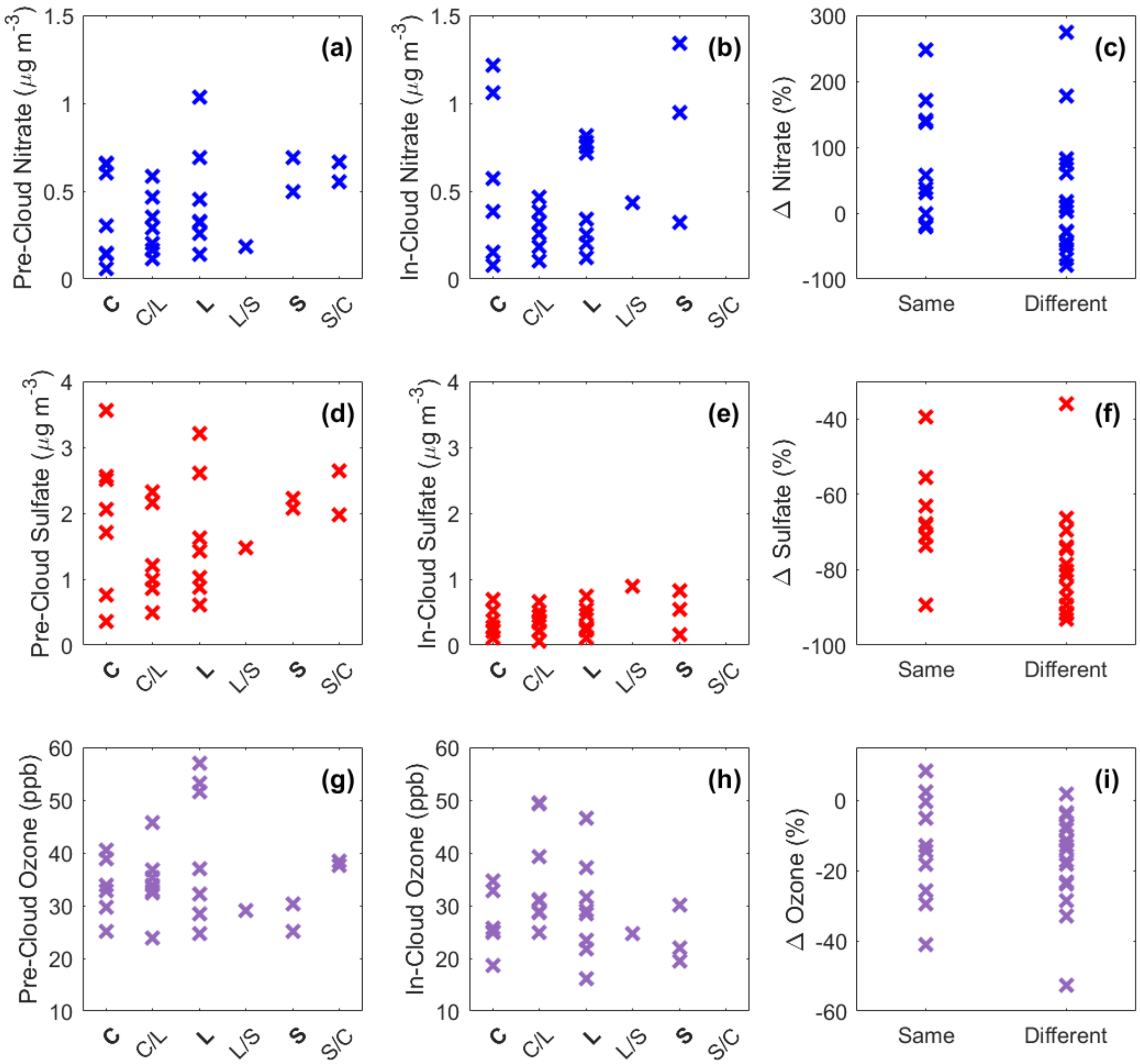

Fig. S7.

(a) Mean nitrate mass concentration for the 26 single-layer pre-cloud events separated by the event back-trajectory classes of coastal northwesterly (C), Los Angeles/Long Beach (L), southerly (S), and mixes of C/L, L/S, and S/C. (b) Same as (a) but for the 26 single-layer in-cloud events. (c) The percent change in mean nitrate mass concentration from pre-cloud to in-cloud separated by events where the pre-cloud and in-cloud back-trajectory classes are the same and events where the pre-cloud and in-cloud back-trajectory classes are different. (d)-(f) Same as (a)-(c) but for mean sulfate mass concentration. (g)-(i) Same as (a)-(c) but for ozone concentration. There is no statistically significant (by t-test) difference in concentrations amongst the back-trajectory class categories or between the same/different categories.

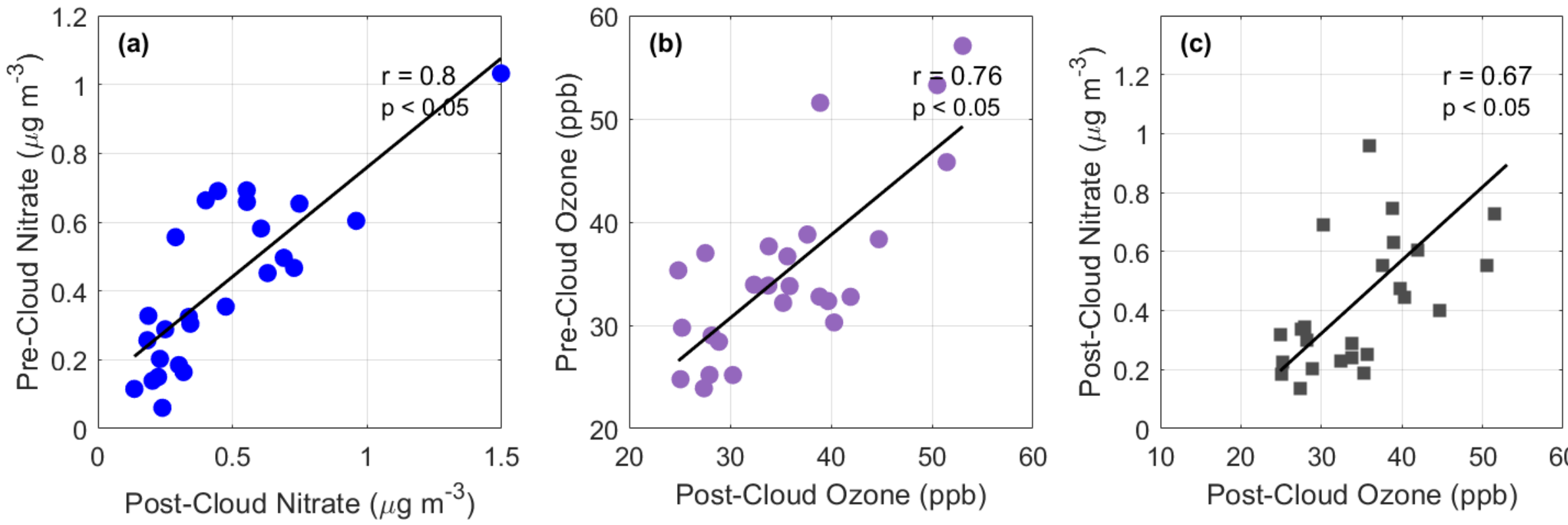

Fig. S8.

Scatter plots showing the 26 single-layer cloud event values of (a) the mean pre-cloud against the mean post-cloud nitrate mass concentrations, (b) the mean pre-cloud against the mean post-cloud nitrate mass concentrations, and (c) the mean post-cloud nitrate mass concentration against the mean post-cloud ozone concentration.

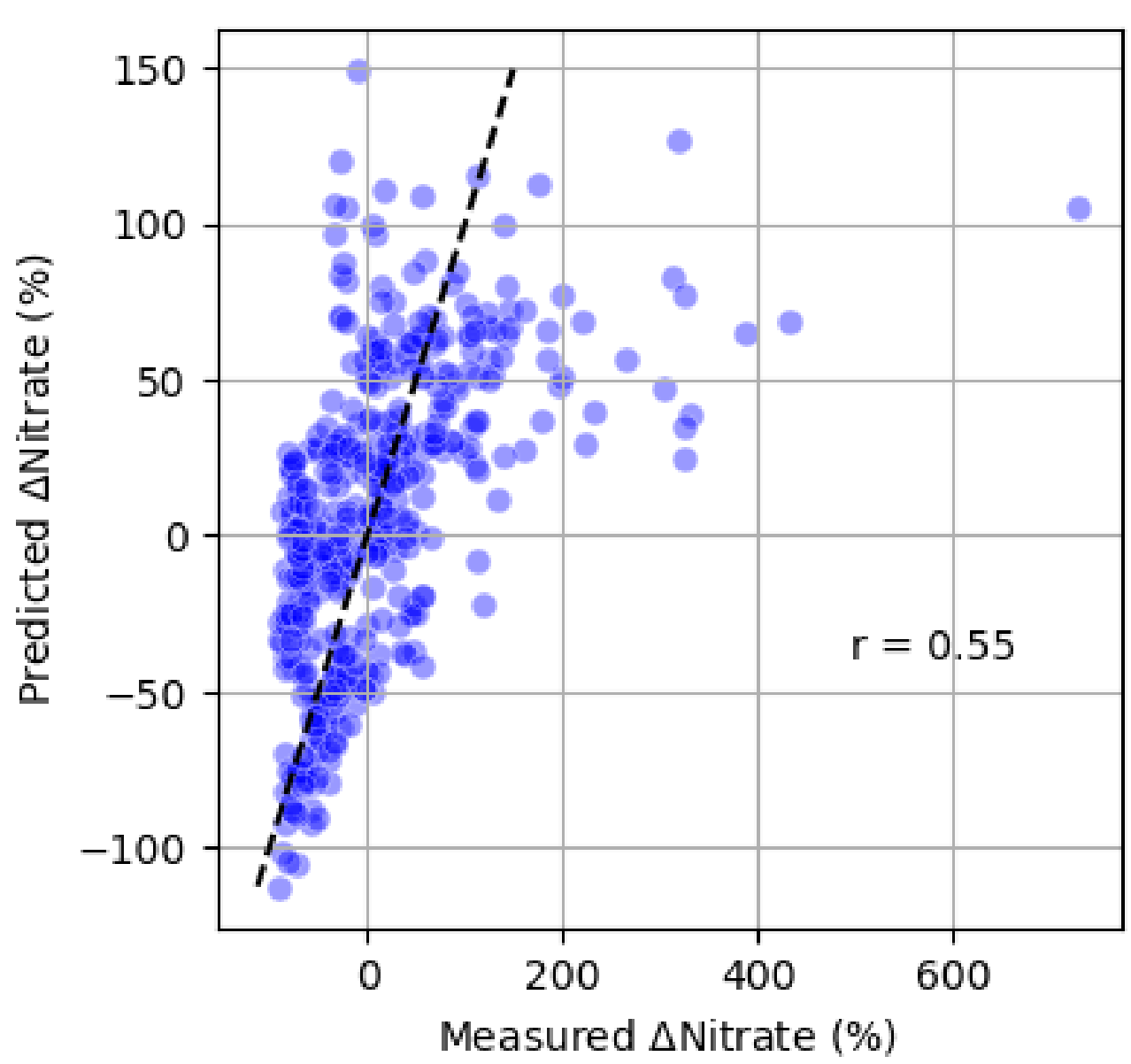

Fig. S9.

Scatter plot of $\Delta Nitrate_{15M}$ predicted by the ridge regression model against $\Delta Nitrate_{15M}$ calculated from observational measurements during the 26 single-layer in-cloud events when all measurements are available (110 hours, 439 points). Markers are semi-transparent to show density. Overlaid is the 1:1 line (dashed black) and the correlation coefficient. The standardized model indicates that $\Delta Nitrate_{15M} = -0.34(Ozone) + 0.23(LWC) + 0.22(Nd) - 0.21(Cloud\ Thickness) + 0.13(Temperature) + 6.2E\text{-}17$ and can explain 30% of the variability (R^2=0.30) in $\Delta Nitrate_{15M}$.

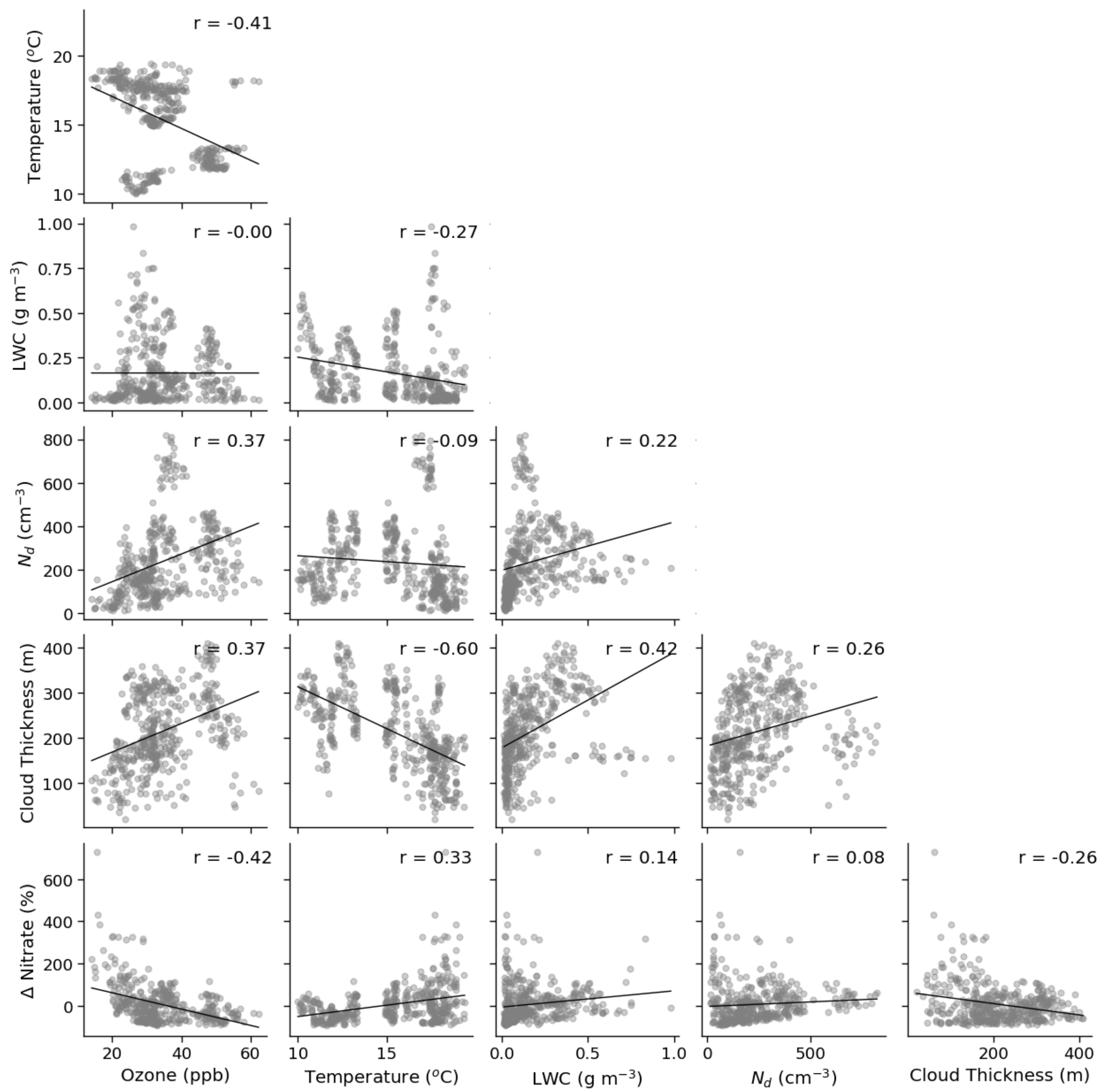

Fig. S10.

Correlation matrix of the 15-minute resolution points where all measurements are available during the 26 single-layer in-cloud events (439 points, 110 hours) for the percent change in nitrate concentration from pre-cloud to in-cloud (Δ Nitrate), in-cloud ozone concentration, in-cloud temperature, liquid water content (LWC), droplet number concentration (Nd), and cloud thickness. All scatter plots are overlaid with the linear regression line and correlation coefficient. Markers are semi-transparent to show density.

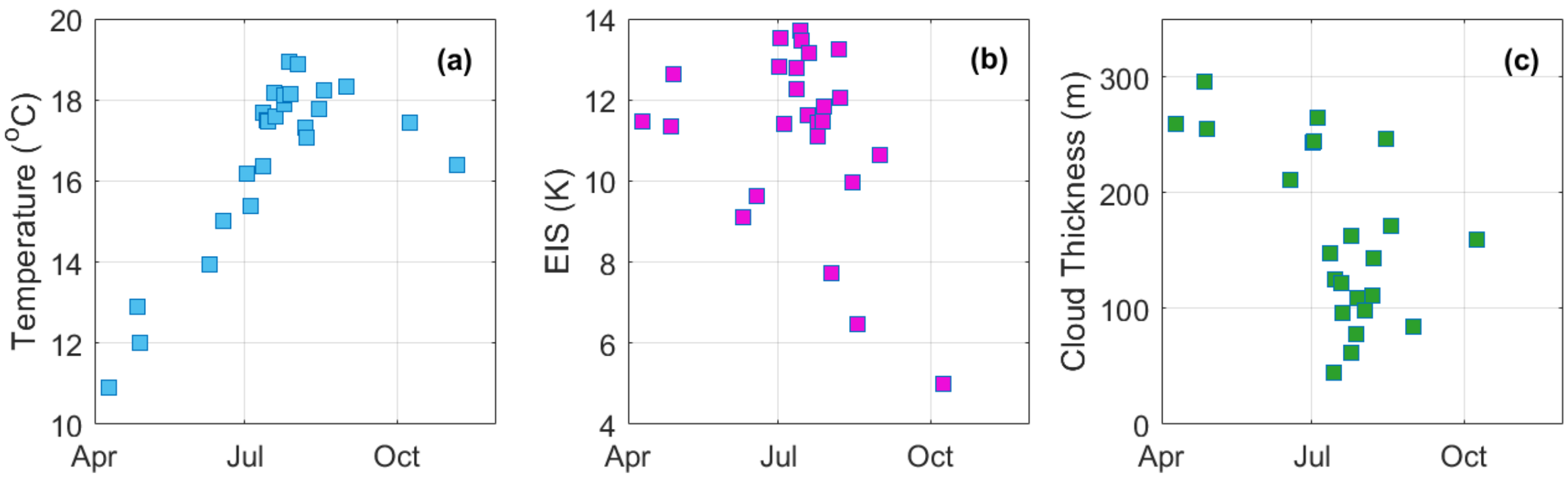

Fig. S11.

Scatter plots of (a) mean temperature, (b) mean estimated inversion strength (EIS), and (c) mean cloud thickness for the 26 single-layer cloud events against the time the cloud event occurred.

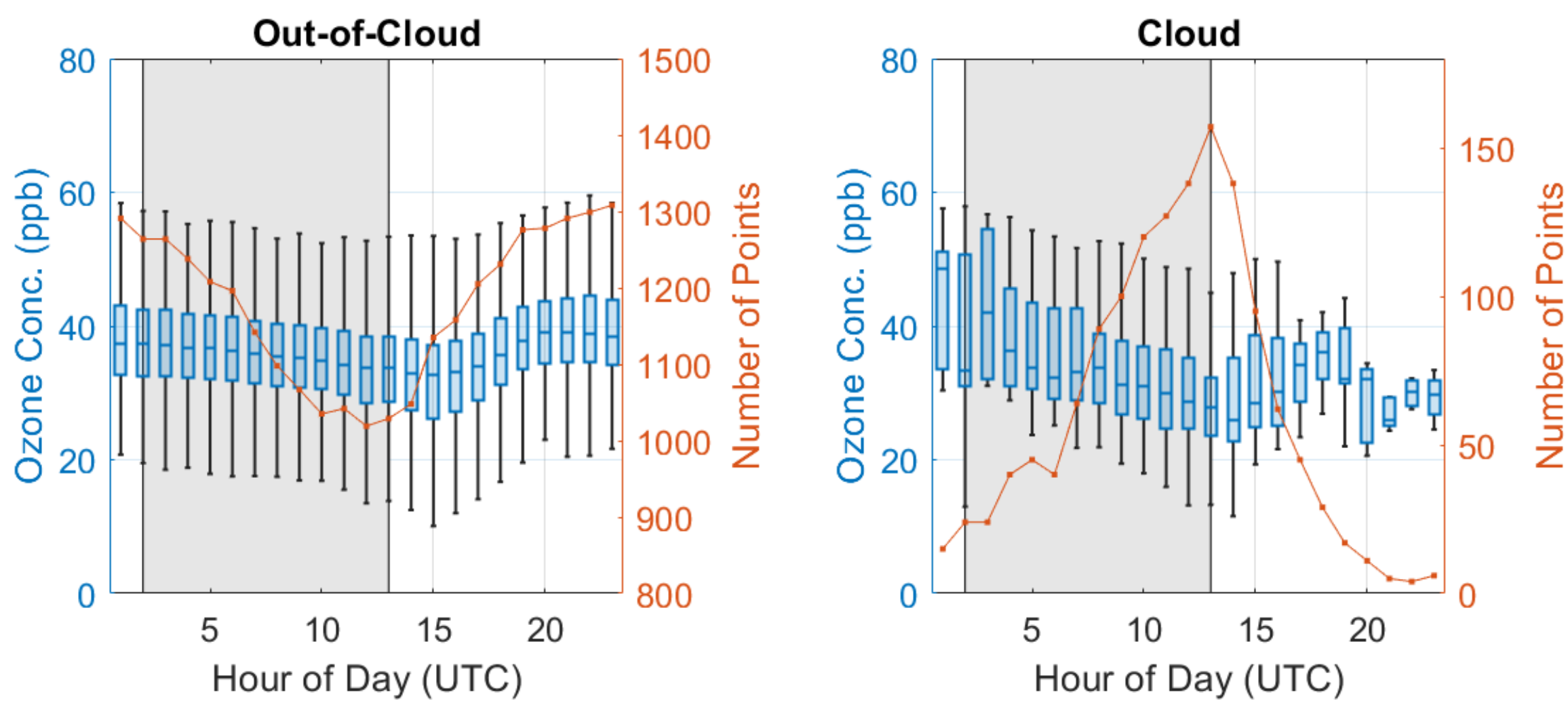

Fig. S12.

Boxcharts of the ozone concentration at Mt. Soledad at 15-min resolution separated by hour of day during out-of-cloud conditions (left) and cloud conditions (right). Overlaid is the number of 15-min points in each hour-of-day group. The shaded region indicates nighttime hours.

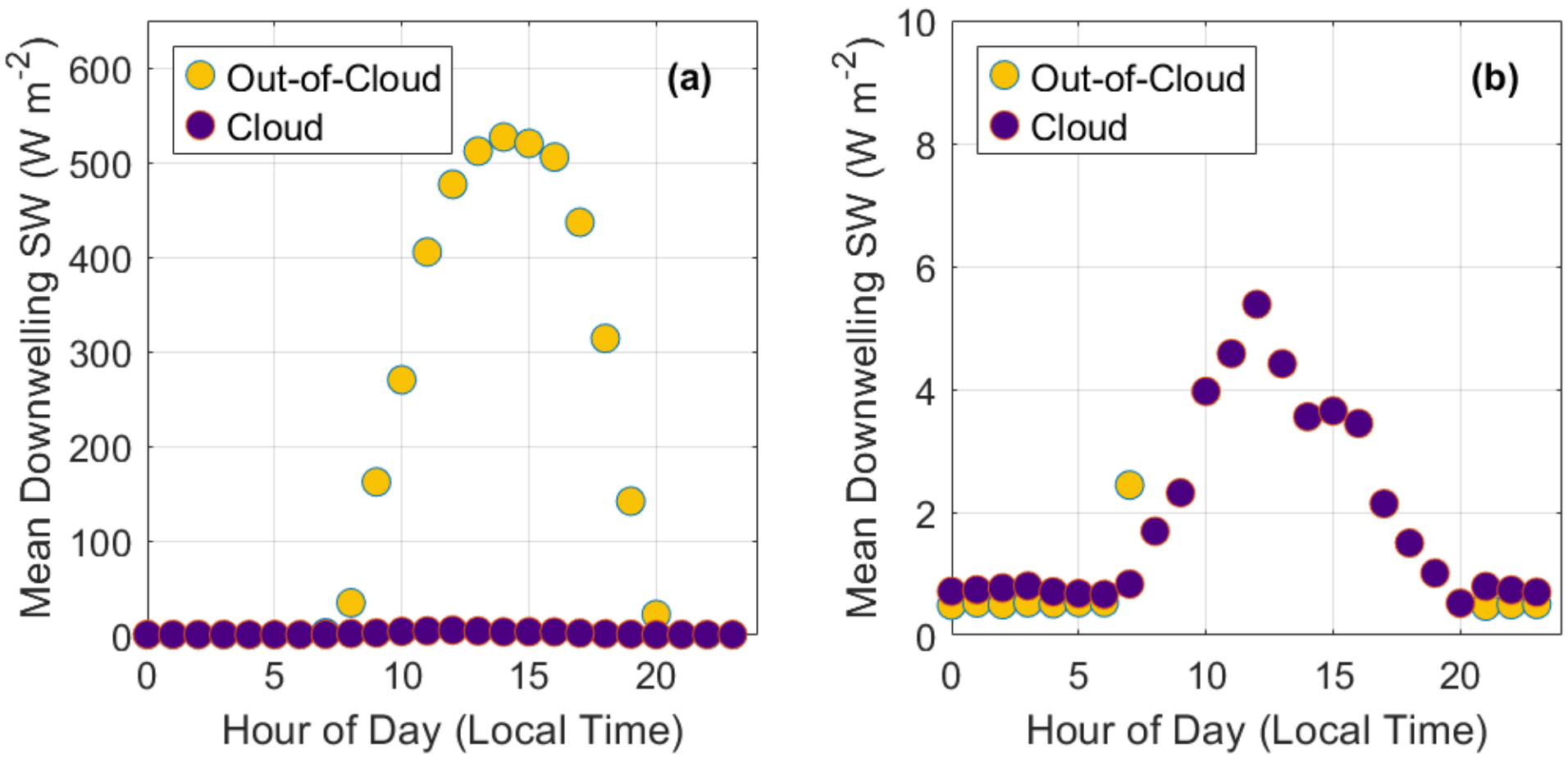

Fig. S13.
(a) Mean downwelling shortwave radiation by hour of day separated for out-of-cloud and cloud conditions. (b) Same as (a) but with a smaller y-axis scale.

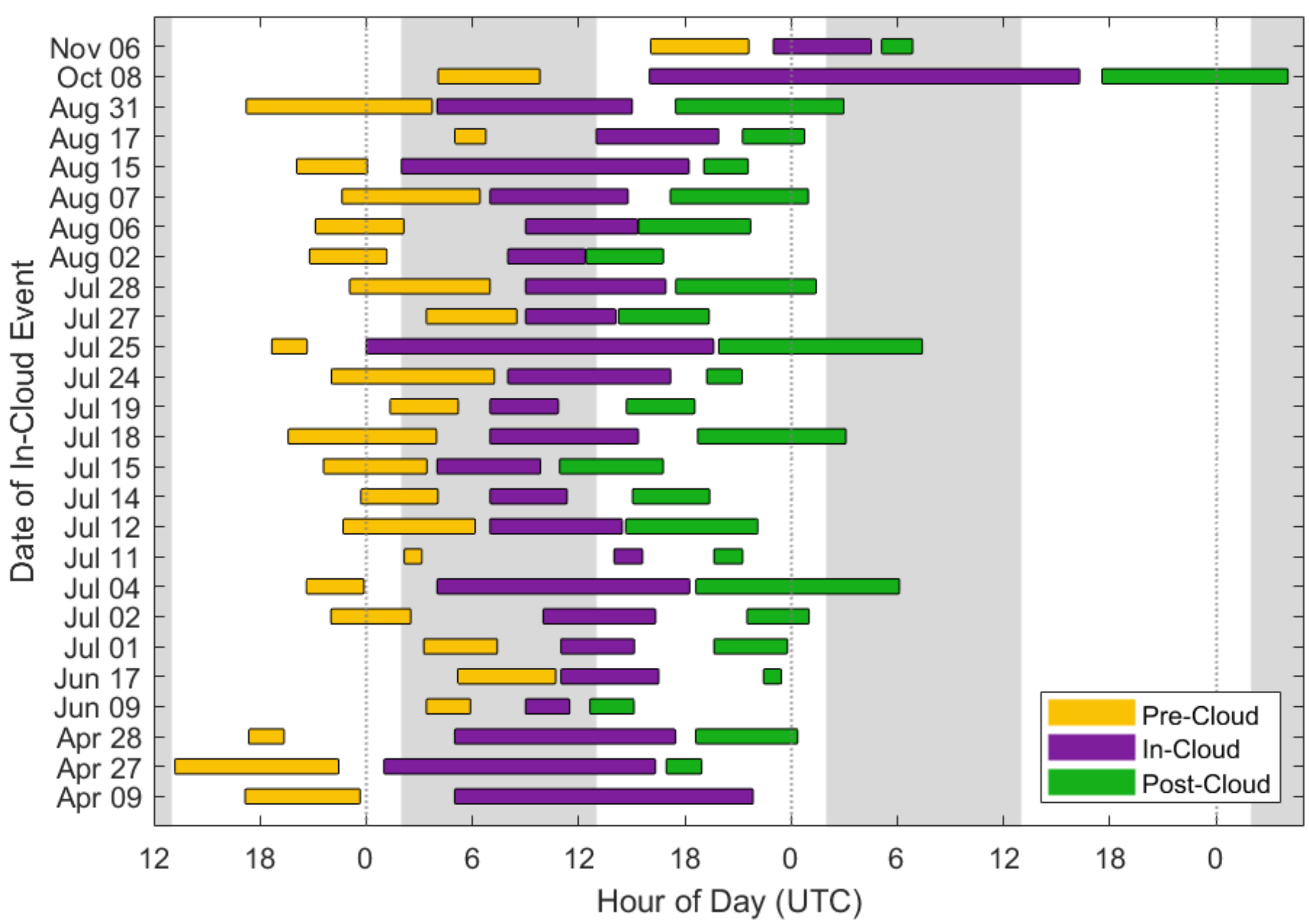

Fig. S14.
Duration plotted by hour of day (x-axis) for the pre-cloud, in-cloud, and post-cloud events for each of the 26 single-layer cloud events (y-axis). Shaded regions indicate nighttime hours.

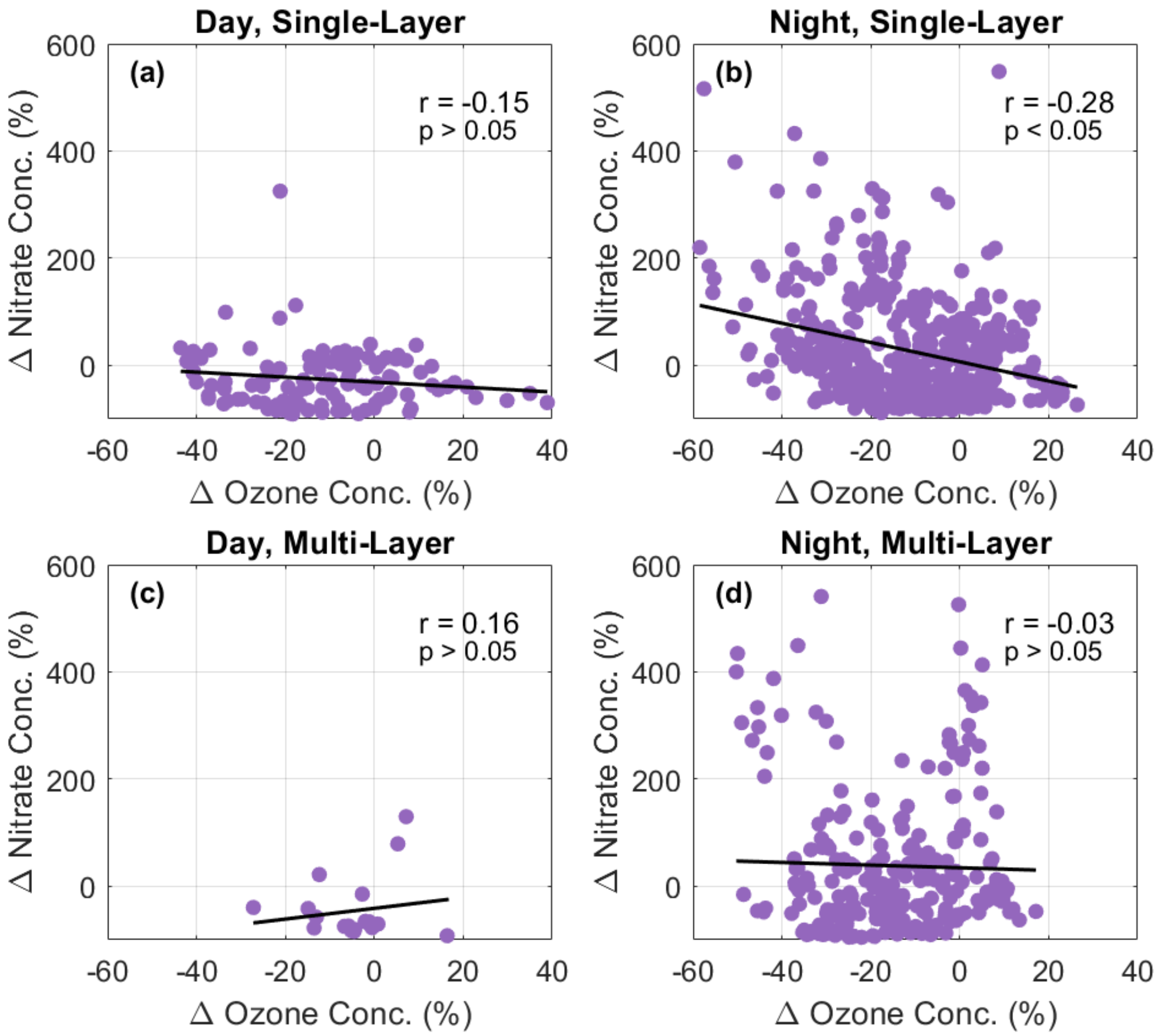

Fig. S15.

The 15-minute (15M) version of the percent change in nitrate mass concentration (i.e. the in-cloud percent change relative to the pre-cloud mean) against the 15M version of the percent change in ozone concentration for (a) during daytime hours for single-layer cloud events, (b) during nighttime hours for single-layer cloud events, (c) during daytime hours for multi-layer cloud events, and (d) during nighttime hours for multi-layer cloud events. Daytime hours are between 1400 and 100 UTC (7AM and 6PM local time) and nighttime hours are between 100 and 1400 UTC (6PM and 7AM local time).

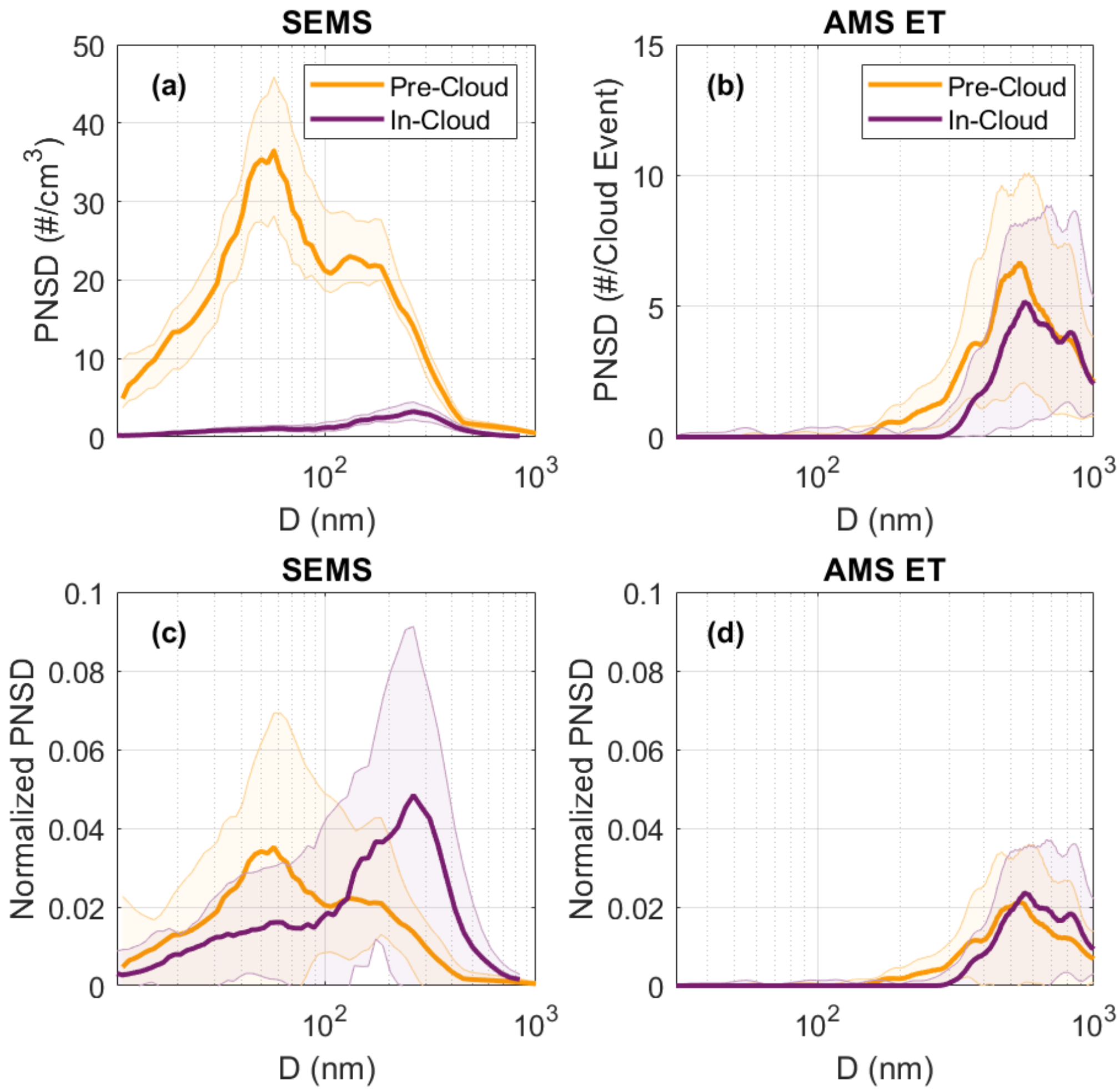

Fig. S16.

(a) Median particle number size distribution (PNSD) measured by the SEMS during the 26 single-layer cloud events. (b) Median PNSD of the sum number of single-particles measured by the AMS during each cloud event for the 26 single-layer cloud events. (c) Same as (a) but normalized by the integral of the median PNSD shown in (a), i.e. normalized by the number concentration. (d) Same as (b) but normalized by the integral of the median PNSD shown in (b), i.e. normalized by the sum of the number of particles measured per cloud event. For all plots, data are separated by pre-cloud and in-cloud periods and shaded regions indicate the 30th and 70th percentiles.

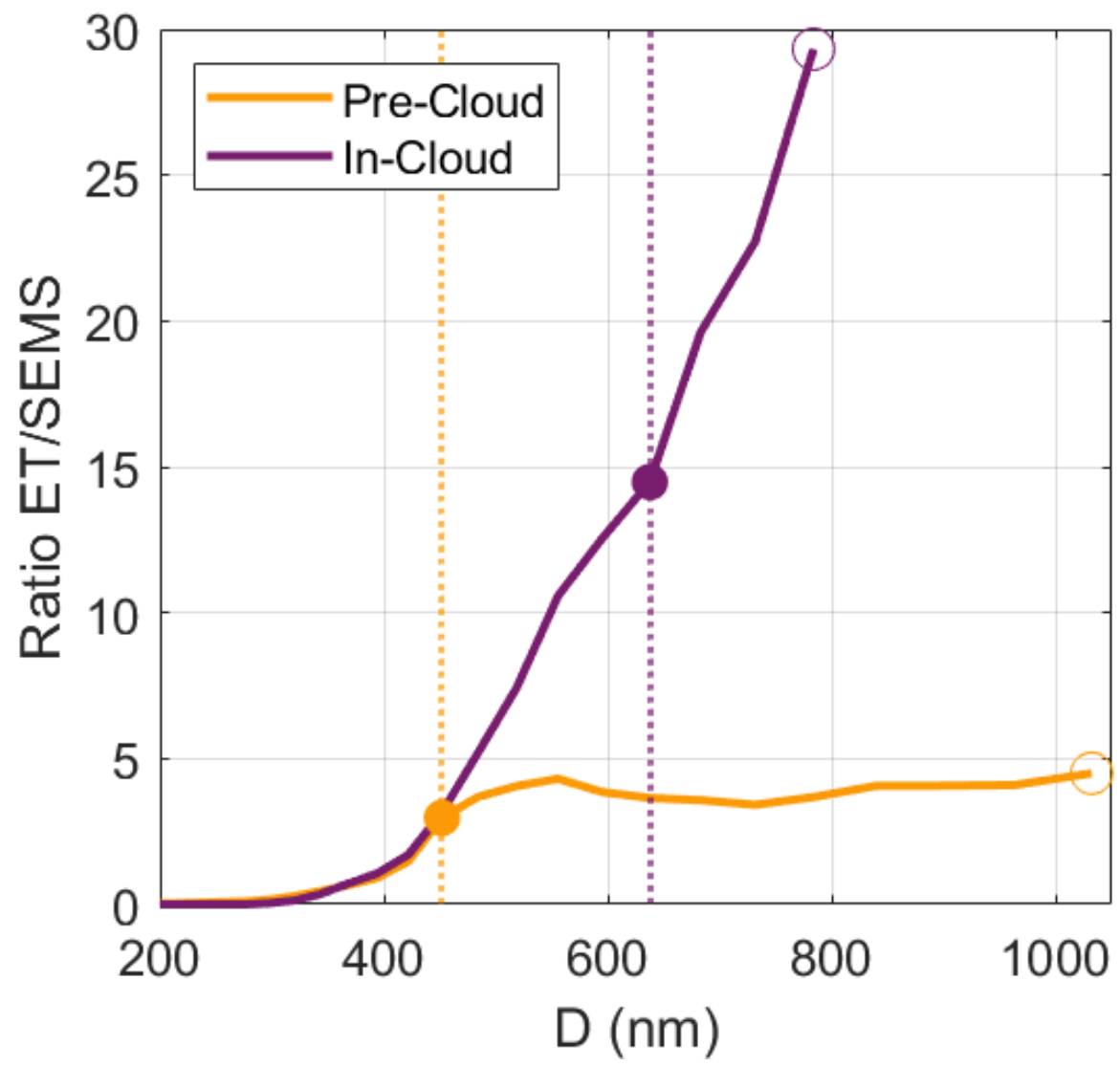

Fig. S17.

Ratio of the median ET PNSD (shown in Figure S2b) to the median SEMS PNSD (shown in Figure S2a) for pre-cloud and in-cloud periods during the 26 single-layer cloud events. Filled dots indicate the fifty-percent diameter (D50) and open dots indicate the maximum value used to determine D50. The in-cloud line ends at ~800 nm due to this being the cutoff size for the SEMS. Pre-cloud measurements used merged SEMS-APS distributions (see Section 2.2) and so the pre-cloud line extends to 1000 nm.

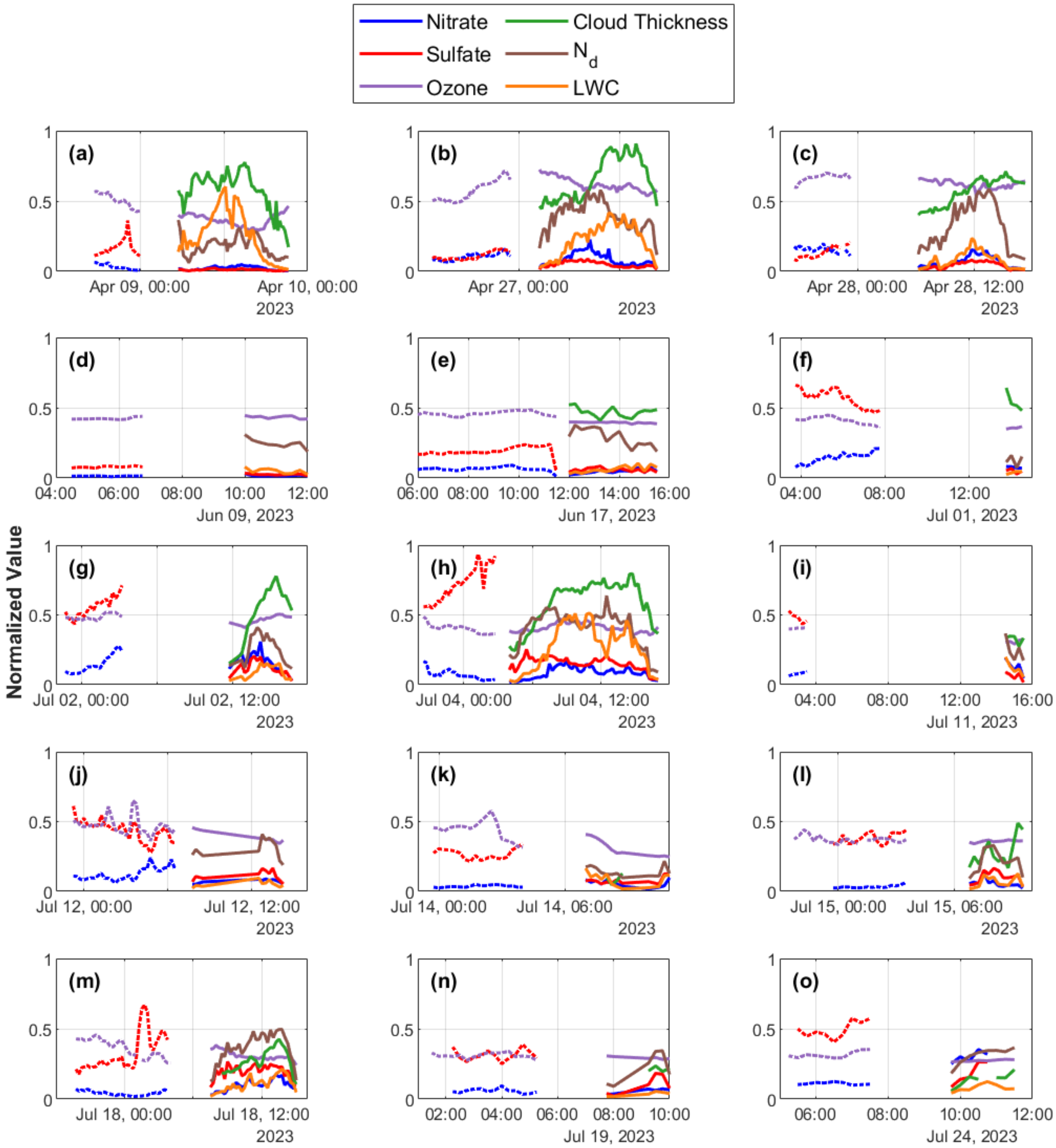

Fig. S18.

Time series of the nitrate mass concentration, sulfate mass concentration, ozone concentration, cloud thickness, droplet number (Nd), and liquid water content (LWC) normalized to the same unitless scale between 0 and 1 (i.e. divided by the absolute maximum value amongst all events) for events 1-15 of 26 single-layer cloud events. Pre-cloud periods are in dashed lines, in-cloud periods are in solid lines (haze conditions are excluded, only cloud conditions are shown. The start and stop times of each cloud event (represented by the letter in the upper left of each subplot) are given in Table S1.

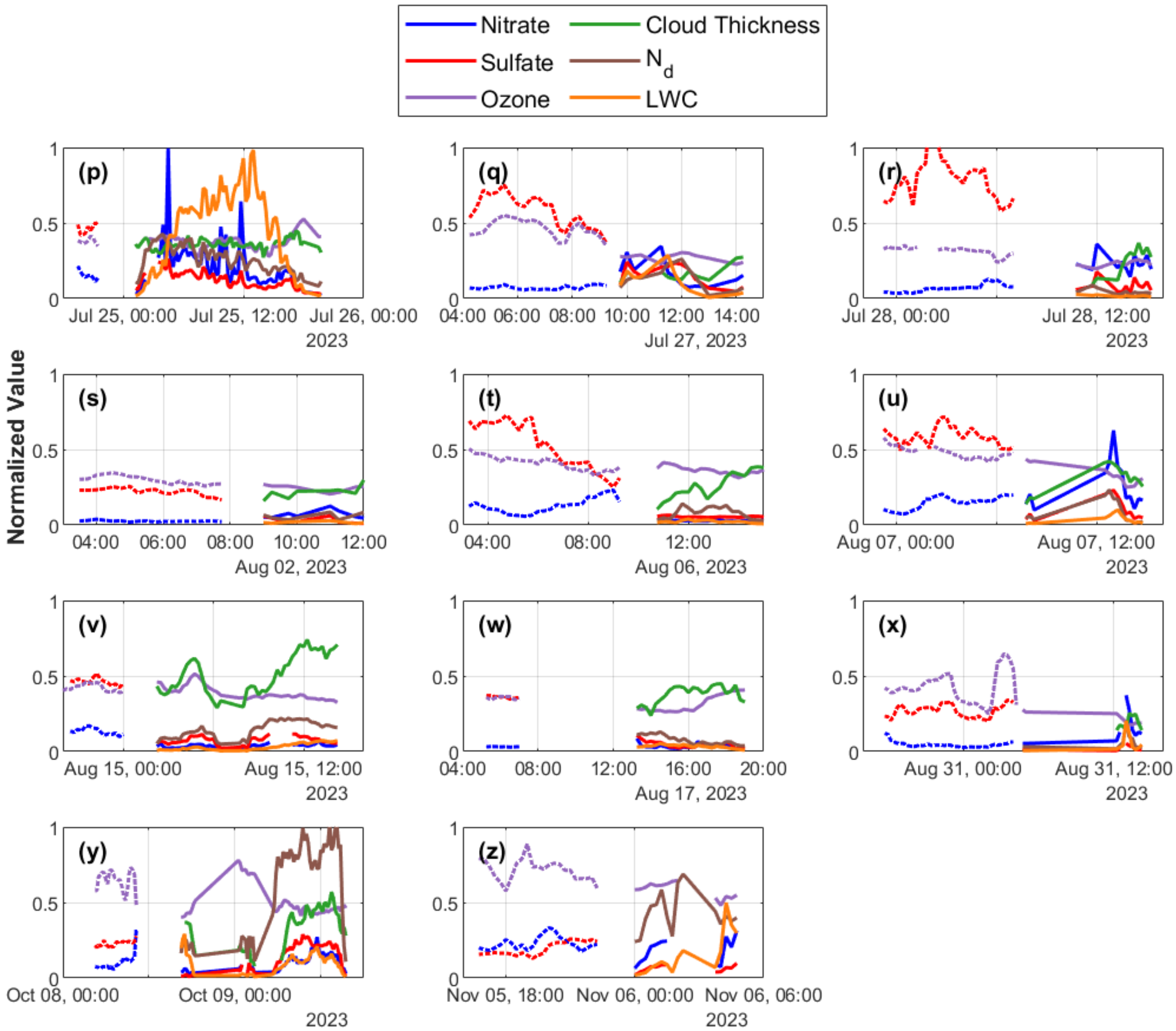

Fig. S19.

Time series of the nitrate mass concentration, sulfate mass concentration, ozone concentration, cloud thickness, droplet number (Nd), and liquid water content (LWC) normalized to the same unitless scale between 0 and 1 (i.e. divided by the absolute maximum value amongst all events) for events 16-26 of 26 single-layer cloud events. Pre-cloud periods are in dashed lines, in-cloud periods are in solid lines (haze conditions are excluded, only cloud conditions are shown). The start and stop times of each cloud event (represented by the letter in the upper left of each subplot) are given in Table S1.

REFERENCES

1. S. E. Bauer, D. Koch, N. Unger, S. M. Metzger, D. T. Shindell, D. G. Streets, Nitrate aerosols today and in 2030: A global simulation including aerosols and tropospheric ozone. *Atmos. Chem. Phys.* **7**, 5043–5059 (2007).

2. G. H. Zhang, X. D. Hu, W. Sun, Y. X. Yang, Z. Y. Guo, Y. Z. Fu, H. C. Wang, S. Z. Zhou, L. Li, M. J. Tang, Z. B. Shi, D. H. Chen, X. H. Bi, X. M. Wang, A comprehensive study about the in-cloud processing of nitrate through coupled measurements of individual cloud residuals and cloud water. *Atmos. Chem. Phys.* **22**, 9571–9582 (2022).

3. E. E. McDuffie, D. L. Fibiger, W. P. Dubé, F. Lopez-Hilfiker, B. H. Lee, J. A. Thornton, V. Shah, L. Jaeglé, H. Y. Guo, R. J. Weber, J. M. Reeves, A. J. Weinheimer, J. C. Schroder, P. Campuzano-Jost, J. L. Jimenez, J. E. Dibb, P. Veres, C. Ebben, T. L. Sparks, P. J. Wooldridge, R. C. Cohen, R. S. Hornbrook, E. C. Apel, T. Campos, S. R. Hall, K. Ullmann, S. S. Brown, Heterogeneous N2O5 uptake during winter: Aircraft measurements during the 2015 WINTER campaign and critical evaluation of current parameterizations. *J. Geophys. Res. Atmos.* **123**, 4345–4372 (2018).

4. S. Crumeyrolle, A. Mensah, A. Khlystov, G. Kos, H. ten Brink, On the importance of nitrate for the droplet concentration in stratocumulus in the North-Sea region. *Atmos. Environ.* **252**, 118278 (2021).

5. Y. C. Chan, M. J. Evans, P. Z. He, C. D. Holmes, L. Jaeglé, P. Kasibhatla, X. Y. Liu, T. Sherwen, J. A. Thornton, X. Wang, Z. Q. Xie, S. T. Zhai, B. Alexander, Heterogeneous nitrate production mechanisms in intense haze events in the North China Plain. *J. Geophys. Res. Atmos.* **126**, e2021JD034688 (2021).

6. W. M. Gong, C. Stroud, L. M. Zhang, Cloud processing of gases and aerosols in air quality modeling. *Atmosphere* **2**, 567–616 (2011).

7. A. Milousis, S. Scholz, H. Fuchs, A. Tsimpidi, V. Karydis, Global perspectives on nitrate aerosol dynamics: A comprehensive sensitivity analysis. *Atmos. Chem. Phys.* **26**, 571–605 (2026).

8. F. Drewnick, J. Schneider, S. S. Hings, N. Hock, K. Noone, A. Targino, S. Weimer, S. Borrmann, Measurement of ambient, interstitial, and residual aerosol particles on a mountaintop site in central Sweden using an aerosol mass spectrometer and a CVI. *J. Atmos. Chem.* **56**, 1–20 (2006).

9. J. Schneider, S. Mertes, D. van Pinxteren, H. Herrmann, S. Borrmann, Uptake of nitric acid, ammonia, and organics in orographic clouds: Mass spectrometric analyses of droplet residual and interstitial aerosol particles. *Atmos. Chem. Phys.* **17**, 1571–1593 (2017).

10. L. K. Berg, C. M. Berkowitz, J. A. Ogren, C. A. Hostetler, R. A. Ferrare, M. K. Dubey, E. Andrews, R. L. Coulter, J. W. Hair, J. M. Hubbe, Y. N. Lee, C. Mazzoleni, J. Olfert, S. R. Springston, Overview of the cumulus humilis aerosol processing study. *Bull. Am. Meteorol. Soc.* **90**, 1653–1668 (2009).

11. R. L. Modini, A. A. Frossard, L. Ahlm, L. M. Russell, C. E. Corrigan, G. C. Roberts, L. N. Hawkins, J. C. Schroder, A. K. Bertram, R. Zhao, A. K. Y. Lee, J. P. D. Abbatt, J. Lin, A. Nenes, Z. Wang, A. Wonaschütz, A. Sorooshian, K. J. Noone, H. Jonsson, J. H. Seinfeld, D. Toom-Sauntry, A. M. Macdonald, W. R. Leaitch, Primary marine aerosol-cloud interactions off the coast of California. *J. Geophys. Res. Atmos.* **120**, 4282–4303 (2015).

12. F. Mattsson, A. Neuberger, L. Heikkinen, Y. Gramlich, M. Paglione, M. Rinaldi, S. Decesari, P. Zieger, I. Riipinen, C. Mohr, Enrichment of organic nitrogen in fog residuals observed in the Italian Po Valley. *Atmos. Chem. Phys.* **25**, 7973–7989 (2025).

13. G. Saliba, D. M. Bell, K. J. Suski, J. D. Fast, D. Imre, G. Kulkarni, F. Mei, J. H. Mülmenstädt, M. Pekour, J. E. Shilling, J. Tomlinson, A. C. Varble, J. Wang, J. A. Thornton, A. Zelenyuk, Aircraft measurements of single particle size and composition reveal aerosol size and mixing state dictate their activation into cloud droplets. *Environ. Sci. Atmos.* **3**, 1352–1364 (2023).

14. Q. H. Lin, G. H. Zhang, L. Peng, X. H. Bi, X. M. Wang, F. J. Brechtel, M. Li, D. H. Chen, P. A. Peng, G. Y. Sheng, Z. Zhou, In situ chemical composition measurement of individual cloud residue particles at a mountain site, southern China. *Atmos. Chem. Phys.* **17**, 8473–8488 (2017).

15. W. R. Leaitch, J. W. Strapp, H. A. Wiebe, K. G. Anlauf, G. A. Isaac, Chemical and microphysical studies of nonprecipitating summer cloud in Ontario, Canada. *J. Geophys. Res. Atmos.* **91**, 11821–11831 (1986).

16. P. H. Daum, T. J. Kelly, J. W. Strapp, W. R. Leaitch, P. Joe, R. S. Schemenauer, G. A. Isaac, K. G. Anlauf, H. A. Wiebe, Chemistry and physics of a winter stratus cloud layer—A case-study. *J. Geophys. Res. Atmos.* **92**, 8426–8436 (1987).

17. K. L. Hayden, A. M. Macdonald, W. Gong, D. Toom-Sauntry, K. G. Anlauf, A. Leithead, S. M. Li, W. R. Leaitch, K. Noone, Cloud processing of nitrate. *J. Geophys. Res. Atmos.* **113**, (2008).

18. G. Prabhakar, B. Ervens, Z. Wang, L. C. Maudlin, M. M. Coggon, H. H. Jonsson, J. H. Seinfeld, A. Sorooshian, Sources of nitrate in stratocumulus cloud water: Airborne measurements during the 2011 E-PEACE and 2013 NiCE studies. *Atmos. Environ.* **97**, 166–173 (2014).

19. S. Crumeyrolle, R. Weigel, K. Sellegri, G. Roberts, L. Gomes, A. Stohl, R. Laj, G. Momboisse, T. Bourianne, V. Puygrenier, F. Burnet, F. Chosson, J. L. Brenguier, J. M. Etcheberry, P. Villani, J. M. Pichon, A. Schwarzenboeck, Airborne investigation of the aerosols-cloud interactions in the vicinity and within a marine stratocumulus over the North Sea during EUCAARI (2008). *Atmos. Environ.* **81**, 288–303 (2013).

20. Z. Wang, M. M. Ramirez, H. Dadashazar, A. B. MacDonald, E. Crosbie, K. H. Bates, M. M. Coggon, J. S. Craven, P. Lynch, J. R. Campbell, M. A. Aghdam, R. K. Woods, H. Jonsson, R. C. Flagan, J. H. Seinfeld, A. Sorooshian, Contrasting cloud composition between coupled and decoupled marine boundary layer clouds. *J. Geophys. Res. Atmos.* **121**, 11679–11691 (2016).

21. L. Q. Hao, S. Romakkaniemi, A. Kortelainen, A. Jaatinen, H. Portin, P. Miettinen, M. Komppula, A. Leskinen, A. Virtanen, J. N. Smith, D. Sueper, D. R. Worsnop, K. E. J. Lehtinen, A. Laaksonen, Aerosol chemical composition in cloud events by high resolution time-of-flight aerosol mass spectrometry. *Environ. Sci. Technol.* **47**, 2645–2653 (2013).

22. G. Betito, E. C. Crosbie, C. A. Hostetler, S. Kirschler, R. H. Moore, T. J. Shingler, M. A. Shook, L. W. Siu, C. Voigt, E. L. Winstead, L. D. Ziemba, A. Sorooshian, On the physicochemical differences between cloud droplet residual particles and below-cloud particles over the Northwest Atlantic. *ACS EST Air* **2**, 2195–2207 (2025).

23. A. G. Allen, A. A. Cardoso, A. G. Wiatr, C. M. D. Machado, W. C. Paterlini, J. Baker, Influence of intensive agriculture on dry deposition of aerosol nutrients. *J. Braz. Chem. Soc.* **21**, 87–97 (2010).

24. J. H. Tsai, K. L. Chang, J. J. Lin, Y. H. Lin, H. L. Chiang, Mass-size distributions of particulate sulfate, nitrate, and ammonium in a particulate matter nonattainment region in southern Taiwan. *J. Air Waste Manage. Assoc.* **55**, 502–509 (2005).

25. V. M. Kerminen, R. E. Hillamo, T. Makela, J. L. Jaffrezo, W. Maenhaut, The physicochemical structure of the Greenland summer aerosol and its relation to atmospheric processes. *J. Geophys. Res. Atmos.* **103**, 5661–5670 (1998).

26. J. T. Newberg, B. M. Matthew, C. Anastasio, Chloride and bromide depletions in sea-salt particles over the northeastern Pacific Ocean. *J. Geophys. Res. Atmos.* **110**, (2005).

27. K. Anlauf, S. M. Li, R. Leaitch, J. Brook, K. Hayden, D. Toom-Sauntry, A. Wiebe, Ionic composition and size characteristics of particles in the Lower Fraser Valley: Pacific 2001 Field Study. *Atmos. Environ.* **40**, 2662–2675 (2006).

28. J. L. Dedrick, L. M. Russell, A. J. I. I. I. Sedlacek, C. A. Kuang, M. A. Zawadowicz, D. Lubin, Aerosol-correlated cloud activation for clean conditions in the tropical Atlantic boundary layer during LASIC. *Geophys. Res. Lett.* **51**, e2023GL105798 (2024).

29. J. L. Dedrick, A. Osawa, L. M. Russell, M. A. Miller, L. Robinson, R. Chang, A. S. Williams, V. Berta, E. Ravichandran, M. Petters, M. Wheeler, S. Han, C. N. Pelayo, N. Maneenoi, D. Lubin, J. Uin, O. Enekwizu, M. Zawadowicz, O. Mayol-Bracero, C. H. Twohy, Quantitative estimates of upwind and onsite particle scavenging and retrieved cloud supersaturations from urban coastal stratocumulus during EPCAPE. *J. Atmos. Sci.* **83**, 1061–1082 (2026).

30. L. M. Russell, J. H. Seinfeld, R. C. Flagan, R. J. Ferek, D. A. Hegg, P. V. Hobbs, W. Wobrock, A. I. Flossmann, C. D. O'Dowd, K. E. Nielsen, P. A. Durkee, Aerosol dynamics in ship tracks. *J. Geophys. Res. Atmos.* **104**, 31077–31095 (1999).

31. R. McVay, B. Ervens, A microphysical parameterization of aqSOA and sulfate formation in clouds. *Geophys. Res. Lett.* **44**, 7500–7509 (2017).

32. N. Maneenoi, L. M. Russell, S. Han, J. L. Dedrick, A. S. Williams, V. Z. Berta, C. Pelayo, M. A. Zawadowicz, A. J. Sedlacek III, I. Silber, M. Thieman, D. Painemal, S. S. P. Shen, Photochemical and cloud and aerosol aqueous contributions to regionally-emitted shipping and biogenic non-sea-salt sulfate aerosol in coastal California. *ACS EST Air* **2**, 648–664 (2025).

33. S. Han, A. S. Williams, L. M. Russell, V. Z. Berta, J. L. Dedrick, C. Pelayo, N. Maneenoi, A. Osawa, I. Silber, D. Zhang, M. A. Zawadowicz, A. J. Sedlacek III, Nearby sites show similar upwind sources and differing semivolatile concentrations in coastal aerosol particles. *ACS EST Air* **2**, 2824–2837 (2025).

34. S. S. Chen, L. L. Xue, M. K. Yau, Hygroscopic seeding effects of giant aerosol particles simulated by the Lagrangian-particle-based direct numerical simulation. *Geophys. Res. Lett.* **48**, e2021GL094621 (2021).

35. Y. J. Tham, Z. Wang, Q. Y. Li, W. H. Wang, X. F. Wang, K. D. Lu, N. Ma, C. Yan, S. Kecorius, A. Wiedensohler, Y. H. Zhang, T. Wang, Heterogeneous N2O5 uptake coefficient and production yield of ClNO2 in polluted northern China: Roles of aerosol water content and chemical composition. *Atmos. Chem. Phys.* **18**, 13155–13171 (2018).

36. Y. Wang, Y. Chen, Z. J. Wu, D. J. Shang, Y. X. Bian, Z. F. Du, S. H. Schmitt, R. Su, G. Gkatzelis, P. Schlag, T. Hohaus, A. Voliotis, K. D. Lu, L. M. Zen, C. S. Zhao, M. R. Alfarra, G. McFiggans, A. Wiedensohler, A. Kiendler-Scharr, Y. H. Zhang, M. Hu, Mutual promotion between aerosol particle liquid water and particulate nitrate enhancement leads to severe nitrate-dominated particulate matter pollution and low visibility. *Atmos. Chem. Phys.* **20**, 2161–2175 (2020).

37. W. R. Leaitch, J. W. Bottenheim, J. W. Strapp, Possible contribution of N2o5 scavenging to Hno3 observed in winter stratiform cloud. *J. Geophys. Res. Atmos.* **93**, 12569–12584 (1988).

38. S. F. Iacobellis, D. R. Cayan, The variability of California summertime marine stratus: Impacts on surface air temperatures. *J Geophys. Res. Atmos.* **118**, 9105–9122 (2013).

39. R. Wood, “CLOUDS AND FOG | Stratus and Stratocumulus,” in *Encyclopedia of Atmospheric Sciences (Second Edition)*, G. R. North, J. Pyle, F. Zhang, Eds. (Academic Press, 2015), pp. 196–200.

40. J. H. Seinfeld, S. N. Pandis, *Atmospheric Chemistry and Physics* (John Wiley & Sons Inc., ed. 3, 2016).

41. M. Trainer, D. D. Parrish, M. P. Buhr, R. B. Norton, F. C. Fehsenfeld, K. G. Anlauf, J. W. Bottenheim, Y. Z. Tang, H. A. Wiebe, J. M. Roberts, R. L. Tanner, L. Newman, V. C. Bowersox, J. F. Meagher, K. J. Olszyna, M. O. Rodgers, T. Wang, H. Berresheim, K. L. Demerjian, U. K. Roychowdhury, Correlation of ozone with noy in photochemically aged air. *J. Geophys. Res. Atmos.* **98**, 2917–2925 (1993).

42. B. Ervens, Modeling the processing of aerosol and trace gases in clouds and fogs. *Chem. Rev.* **115**, 4157–4198 (2015).

43. L. Kaser, E. G. Patton, G. G. Pfister, A. J. Weinheimer, D. D. Montzka, F. Flocke, A. M. Thompson, R. M. Stauffer, H. S. Halliday, The effect of entrainment through atmospheric boundary layer growth on observed and modeled surface ozone in the Colorado Front Range. *J. Geophys. Res. Atmos.* **122**, 6075–6093 (2017).

44. J. A. Neuman, M. Trainer, K. C. Aikin, W. M. Angevine, J. Brioude, S. S. Brown, J. A. de Gouw, W. P. Dube, J. H. Flynn, M. Graus, J. S. Holloway, B. L. Lefer, P. Nedelec, J. B. Nowak, D. D. Parrish, I. B. Pollack, J. M. Roberts, T. B. Ryerson, H. Smit, V. Thouret, N. L. Wagner, Observations of ozone transport from the free troposphere to the Los Angeles basin. *J. Geophys. Res. Atmos.* **117**, (2012).

45. J. Flynn, B. Lefer, B. Rappenglück, M. Leuchner, R. Perna, J. Dibb, L. Ziemba, C. Anderson, J. Stutz, W. Brune, X. R. Ren, J. Q. Mao, W. Luke, J. Olson, G. Chen, J. Crawford, Impact of clouds and aerosols on ozone production in Southeast Texas. *Atmos. Environ.* **44**, 4126–4133 (2010).

46. Y. Miyazaki, K. Kita, Y. Kondo, M. Koike, M. Ko, W. Hu, S. Kawakami, D. R. Blake, T. Ogawa, Springtime photochemical ozone production observed in the upper troposphere over east Asia. *J. Geophys. Res. Atmos.* **107**, BIB 2-1–BIB 2-14 (2002).

47. R. Vautard, P. Yiou, G. J. van Oldenborgh, Decline of fog, mist and haze in Europe over the past 30 years. *Nat. Geosci.* **2**, 115–119 (2009).

48. X. J. Shen, J. Y. Sun, X. Y. Zhang, Y. M. Zhang, L. Zhang, H. C. Che, Q. L. Ma, X. M. Yu, Y. Yue, Y. W. Zhang, Characterization of submicron aerosols and effect on visibility during a severe haze-fog episode in Yangtze River Delta, China. *Atmos. Environ.* **120**, 307–316 (2015).

49. S. Kim, C. Rickard, J. Hernandez-Vazquez, D. Fernandez, Early night fog prediction using liquid water content measurement in the Monterey Bay Area. *Atmosphere* **13**, 1332 (2022).

50. R. G. Eldridge, Mist—Transition from haze to fog. *Bull. Am. Meteorol. Soc.* **50**, 422–427 (1969).

51. T. Shingler, S. Dey, A. Sorooshian, F. J. Brechtel, Z. Wang, A. Metcalf, M. Coggon, J. Mülmenstädt, L. M. Russell, H. H. Jonsson, J. H. Seinfeld, Characterisation and airborne deployment of a new counterflow virtual impactor inlet. *Atmos. Meas. Tech.* **5**, 1259–1269 (2012).

52. W. W. Hu, P. Campuzano-Jost, D. A. Day, P. Croteau, M. R. Canagaratna, J. T. Jayne, D. R. Worsnop, J. L. Jimenez, Evaluation of the new capture vapourizer for aerosol mass spectrometers (AMS) through laboratory studies of inorganic species. *Atmos. Meas. Tech.* **10**, 2897–2921 (2017).

53. C. Knote, D. Brunner, H. Vogel, J. Allan, A. Asmi, M. Äijälä, S. Carbone, H. D. van der Gon, J. L. Jimenez, A. Kiendler-Scharr, C. Mohr, L. Poulain, A. S. H. Prévôt, E. Swietlicki, B. Vogel, Towards an online-coupled chemistry-climate model: Evaluation of trace gases and aerosols in COSMO-ART. *Geosci. Model Dev.* **4**, 1077–1102 (2011).

54. J. Fast, A. C. Aiken, J. Allan, L. Alexander, T. Campos, M. R. Canagaratna, E. Chapman, P. F. DeCarlo, B. de Foy, J. Gaffney, J. de Gouw, J. C. Doran, L. Emmons, A. Hodzic, S. C. Herndon, G. Huey, J. T. Jayne, J. L. Jimenez, L. Kleinman, W. Kuster, N. Marley, L. Russell, C. Ochoa, T. B. Onasch, M. Pekour, C. Song, I. M. Ulbrich, C. Warneke, D. Welsh-Bon, C. Wiedinmyer, D. R. Worsnop, X. Y. Yu, R. Zaveri, Evaluating simulated primary anthropogenic and biomass burning organic aerosols during MILAGRO: Implications for assessing treatments of secondary organic aerosols. *Atmos. Chem. Phys.* **9**, 6191–6215 (2009).

55. V. Berta, S. Han, A. Osawa, J. Dedrick, N. Maneenoi, L. M. Russell, "Aerosol quantification products at Mt. Soledad and Scripps Pier during EPCAPE 2023–24," in *Aerosol Microphysics and Chemical Measurements at Mt. Soledad and Scripps Pier during the Eastern Pacific Cloud Aerosol Precipitation Experiment (EPCAPE) from February 2023 to February 2024* (UC San Diego Library Digital Collections, 2025).

56. A. K. Y. Lee, M. D. Willis, R. M. Healy, T. B. Onasch, J. P. D. Abbatt, Mixing state of carbonaceous aerosol in an urban environment: Single particle characterization using the soot particle aerosol mass spectrometer (SP-AMS). *Atmos. Chem. Phys.* **15**, 1823–1841 (2015).

57. C. Adam, Pollution Data, edited by San Diego County Air Pollution Control District (2023). https://sdapcd.org/content/sdapcd/air-quality.html.

58. R. Wood, C. S. Bretherton, On the relationship between stratiform low cloud cover and lower-tropospheric stability. *J. Climate* **19**, 6425–6432 (2006).

59. S. S. Shen, G. R. North, *Statistics and Data Visualization in Climate Science with R and Python* (Cambridge University Press, 2023).

60. R. X. Ward, H. D. Baliaka, B. C. Schulze, G. H. Kerr, J. D. Crounse, S. Hasheminassab, R. Bahreini, A. M. Dillner, A. Russell, N. L. Ng, P. O. Wennberg, R. C. Flagan, J. H. Seinfeld, Poorly quantified trends in ammonium nitrate remain critical to understand future urban aerosol control strategies. *Sci. Adv.* **11**, eadt8957 (2025).

61. S. L. Lewis, L. M. Russell, J. A. McKinsey, W. J. Harris, Small contributions of dust to PM2.5 and PM10 concentrations measured downwind of Oceano Dunes. *Atmos. Environ.* **294**, 119515 (2023).

62. C. Li, R. Martin, A. van Donkelaar, J. L. Jimenez, Q. Zhang, J. R. Turner, X. Liu, M. Rowe, J. Meng, W. Y. Yu, G. D. Thurston, Estimates of submicron particulate matter (PM1) concentrations for 1998-2022 across the contiguous USA: Leveraging measurements of PM1 with nationwide PM25 component data. *Lancet Planet. Health* **9**, e491–e502 (2025).

63. P. D. Hien, V. T. Bac, N. T. H. Thinh, H. L. Anh, D. D. Thang, N. T. Nghia, A comparison study of chemical compositions and sources of PM1.0 and PM2.5 in Hanoi. *Aerosol Air Qual. Res.* **21**, 210056 (2021).

64. Z. J. Li, W. Q. Xu, W. Zhou, L. Lei, J. X. Sun, B. You, Z. F. Wang, Y. L. Sun, Insights into the compositional differences of PM1 and PM2.5 from aerosol mass spectrometer measurements in Beijing, China. *Atmos. Environ.* **301**, 119709 (2023).
